# Effect of temperature on Sr and Cs incorporation and mass transport mechanisms in geopolymer cement wasteforms

**DOI:** 10.1039/d6ta01864e

**Published:** 2026-09-02

**Authors:** Charlotte Nevin, Daniel A. Geddes, Martin C. Stennett, Tom J. Wilkinson, Dinu Iuga, Brant Walkley

**Affiliations:** a School of Chemical, Materials, and Biological Engineering, University of Sheffield UK b.walkley@sheffield.ac.uk; b Department of Physics, University of Warwick Coventry UK

## Abstract

Geopolymer wasteforms are a promising alternative to Portland cement for immobilisation of intermediate-level radioactive waste, particularly caesium-137 and strontium-90. Successful implementation relies on ensuring wasteform stability and radionuclide retention under elevated temperatures expected in a geological disposal facility. This study evaluates radionuclide retention and mass transport mechanisms of Cs- and Sr-loaded geopolymers leached at elevated temperatures (35, 50, and 90 °C). Monolithic leach tests confirmed that while Cs consistently exhibits a significantly larger Cumulative Fraction Leached compared with Sr across all temperatures, all geopolymers maintained acceptable leachability indices (Li > 6). Increasing the temperature to 90 °C resulted in a pronounced increase in release rates, with Cs release nearly tripling and Sr release increasing by an order of magnitude, primarily due to accelerated kinetics and increased solubility of secondary phases such as SrCO_3_ at elevated temperatures. Micro- and nanostructural analysis (XRD, FTIR, NMR) confirmed that the K–A–S–H gel framework remained stable, suggesting that bulk structural integrity is not compromised by these temperatures. Mass transport modelling revealed that Cs and Sr release is dominated by a complex, multi-parametric combination of diffusion and surface exchange kinetics (DSEM), with diffusion becoming increasingly dominant at higher temperatures. However, the temperature dependency does not conform to the Arrhenius principle, suggesting the process is governed by multiple overlapping mechanisms rather than a single activation energy across the investigated temperature range. Together, these findings show that geopolymers exhibit excellent thermal stability and maintain superior radionuclide retention performance compared to conventional cementitious systems under simulated repository temperature extremes.

## Abstract 2

Geopolymer wasteforms are a promising alternative to Portland cement for immobilisation of intermediate-level radioactive waste, particularly caesium-137 and strontium-90.

## Graphical abstract

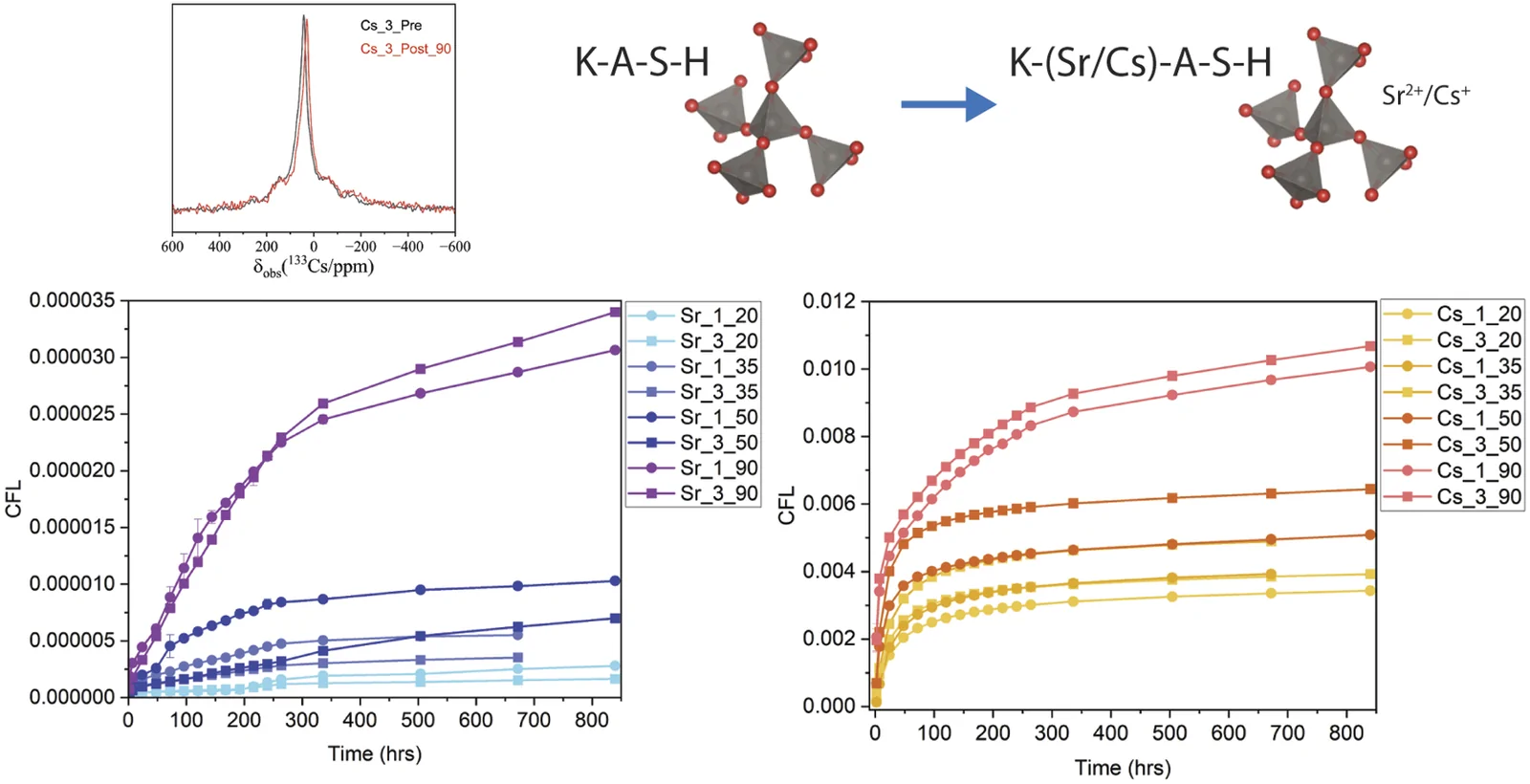

## 1 Introduction

Radioactive waste arising from nuclear activity is hazardous and requires conditioning and disposal to protect humans and the environment. Caesium-137 and strontium-90, two abundant fission products, pose significant challenges and are particularly prevalent in radioactive waste streams. Both radionuclides exhibit high radiotoxicity and an ability to act analogously to elements vital to the functioning of the human body, making them extremely problematic.^1^

At present, intermediate level wastes (ILW), such as Cs-137 and Sr-90, and low level wastes (LLW) are encapsulated in blended Portland cement-blast furnace slag blends, specifically formulated and developed on site at Sellafield in the UK.^2^ These materials offer industrial maturity, security of supply, high throughput, and low cost.^3^ However, issues such as Cs mobilisation,^2,4^ high leaching rates, and supply chain disruptions necessitate a drive for developing alternative solutions.

Geopolymers comprise a structurally disordered, highly cross-linked aluminosilicate gel ((N, K)–A–S–H where Na^+^ and K^+^ are the most common cations).^2^ The structure is pseudo-zeolitic, and the charge-balancing cations balance the negative overall charge arising from the Al^3+^ ion tetrahedral coordination.^5^ The cations can be substituted with other ions (such as Cs^+^ or Sr^2+^) if it is energetically favourable, creating the possibility of chemically binding radionuclides.^2,6^ Geopolymer cements have been highlighted as a potential alternative to PC/BFS cements for the disposal of certain radioactive wastes due to their advantageous performance, *e.g.* lower leaching rates, immobilisation capabilities, and improved durability in extreme conditions.^3^

In the United Kingdom, all wasteforms will ultimately be disposed of in a repository, either in a deep Geological Disposal Facility (GDF) or near-surface repository, dependent on its waste classification.^7^ These repositories will be subjected to extreme conditions due to the handling of radioactive waste. One such condition will be fluctuating temperature^8^ radiogenic heating and other, non-radiogenic mechanisms such as corrosion of waste or microbial degradation of materials.^9^ It is predicted that the repository will experience five key phases of implementation with varying predicted temperatures; emplacement (30–40 °C), care and maintenance (30–40 °C), short-term backfill (80–90 °C), long-term backfill (50 °C), and post-closure (35–45 °C).^8^

Previous work by the authors built on work by Cote, El-Kamash, and Abdel-Rahman in assessing the mass transport of cementitious wasteforms by studying experimental releases through leaching experiments^10,11^ and fitting models built from first principle mass transport.^12–16^ This work found that mass transport was complex and multi-parametric, dominated by diffusion and surface exchange/reaction kinetics.^17^ Building on this work, it is essential to evaluate how temperature affects the performance and mass transport mechanisms of geopolymer cement wasteforms. To address this, Cs- and Sr-loaded geopolymer wasteforms were subjected to monolithic leach testing at repository-relevant temperatures (35, 50, and 90 °C), with radionuclide release rates quantified and interpreted by mass transport modelling. Additionally, structural characterisation was undertaken to determine whether any changes in release behaviour were associated with changes in the geopolymer matrix. This study will provide valuable insights into these effects, ensuring long-term durability and stability under repository-relevant conditions, ultimately strengthening the safety case for geopolymers for radioactive waste disposal.

## 2 Experimental methods

### 2.1 Sample preparation

Geopolymer gels were produced by the reaction between a metakaolin precursor (MetaMax, Lawrence Industries, with composition determined by X-ray fluorescence as in Table 1) and a potassium silicate activating solution (PQ-silicates, solution modulus SiO_2_/K_2_O = 2.2. The solution modulus was reduced to 1 through mixing with potassium hydroxide powder (AnalaR 99 wt%). Stoichiometry was designed to obtain a nominal composition as follows: K_2_O/Al_2_O_3_ = 1, SiO_2_/Al_2_O_3_ = 3, H_2_O/K_2_O = 13. Samples containing caesium and strontium were prepared by mixing 1 or 3 wt% Sr(OH)_2_ or CsOH with the metakaolin prior to addition of activating solution. Samples were subsequently mixed for 10 minutes at 1200 rpm using a high shear mixer to achieve homogeneity, cast in sealed containers, and cured at 20 ± 2 °C for 28 days.

**Table 1:** Metakaolin chemical composition (wt%) as determined by X-ray fluorescence. LOI = loss on ignition at 1000 °C

SiO_2_	Al_2_O_3_	K_2_O	Na_2_O	MgO	TiO_2_	Fe_2_O_3_	Other	LOI
52.5	44.5	0.2	0.2	<0.05	1.3	0.4	0.2	0.6

A conservative loading of 1 wt% was selected to ensure homogeneous dispersion within the metakaolin geopolymer matrix and to preserve sample integrity for mechanistic characterisation; several recent mechanistic studies adopt comparable conservative loadings for the same reason.^18–20^ A moderate loading of 3 wt% was also investigated to probe stronger effects of cation incorporation on mass transport and microstructure, and lies within the loading ranges employed in other experimental studies (for example Mukiza *et al.* used 4 wt% simulants^21^). The selection of these loadings follows established wasteform development practice where achievable solid loadings are balanced against mechanical performance and workability.

After curing, samples were removed from sealed containers and formed into cylinders (13 mm length, 14 mm diameter) using a diamond-tipped slow saw. The ends were sealed to prevent axial mass transfer. Samples underwent a leach test according to a modified ASTM C1308 standard, wherein the monolithic samples were fully submerged in deionised water which was sampled and fully replaced at 2 hours, 7 hours, 24 hours, every day until 11 days, 14 days, 21 days, 28 days, and 35 days. After last time point, the monoliths were removed from solution and submerged in isopropanol to remove any bound water through ion exchange to prevent further reaction. The samples were then prepared for characterisation; the solid phase samples were prepared for characterisation by hand grinding with a pestle and mortar until a talc-like consistency was reached.

### 2.2 Solid-state characterisation

#### 2.2.1 X-ray diffraction (XRD)

A Panalytical X'Pert^3^ Powder X-ray Diffractometer with Cu Kα radiation, a nickel filter, a step size of 0.020°, and a count time of 1 s per step was used to obtain data across a 2*θ* range of 5–70°. To reduce diffracted background intensity at low angles, an anti-scatter blade was used with an incident beam divergence of 1.0 mm. Also, within the diffracted beam, a 2.5° Soller slit was used. Peak analysis and identification was performed using Diffrac.EVA software.

#### 2.2.2 Fourier transform infrared (FTIR) spectroscopy

ATR-FTIR spectra were measured across a range of 400 to 4000 cm^−1^ using a Thermo Fischer Nicolet iS 5 Spectrometer scanning 64 times at a resolution of 16 cm^−1^ and a zinc selenide crystal. The background was always measured before the first sample and automatically removed from the spectrum. Plots are generated with transmission normalised between [0, 1] to allow comparison between spectra.

#### 2.2.3 Nuclear magnetic resonance (NMR) spectroscopy

Solid state single pulse ^29^Si and ^27^Al magic angle spinning (MAS) and cross polarisation (CP) ^1^H–^29^Si MAS NMR data were obtained to examine the local structure around atomic nuclei. Spectra were acquired on a Bruker Avance III HD 500 spectrometer at 11.7 T (*B*_0_) using a 4.0 mm dual resonance CP/MAS probe with a Larmor frequency of 99.35 MHz for ^29^Si and 130.32 MHz for ^27^Al. ^27^Al MAS spectra were acquired using a 1.7 µs non-selective (π/2) excitation pulse, a measured 10 s relaxation delay, spinning at 12.5 kHz, and a total of 128 scans. ^29^Si MAS spectra were aquired using a 5.5 µs non-selective (π/2) excitation pulse, a measured 90 s relaxation delay, spinning at 12.5 kHz with a total of 256 scans. CP MAS spectra were acquired with a ^29^Si 1.7 µs non-selective (π/2) pulse width, an initial ^1^H 2.5 µs non-selective (π/2) pulse width, a recycle delay of 1.5 s, a Hartmann–Hahn contact period of 1.7 ms, a spinning frequency of 12.5 kHz, and a total of 10 240 scans collected. Additionally, a nominal ^1^H decoupling field strength of 80 kHz was employed during acquisition.^2^

High-field solid state ^39^K MAS NMR data were acquired at 20.0 T (*ν* 0 = 39.67 MHz) using a Bruker Avance Neo 850 spectrometer with a Bruker 4.0 mm HX MAS probe, which enabled a spinning rate of 14 kHz to be implemented. Pulse calibration and chemical shift referencing for all 39 K data were achieved using KCl (s) (*δ*_iso_ = 47.8 ppm) as a secondary reference to the IUPAC primary reference of 0.1 M KCl (aq) (*δ*_iso_ = 0 ppm). A ‘non-selective’ π/2 pulse of 12 µs was measured allowing for a ‘selective’ 4 µs π/3 to be implemented. Spectra were acquired using a Hahn echo pulse sequence (π/2–*τ*–π–acquire) using a measured relaxation delay of 0.1 s and acquiring a total of 480 000 transients per spectra.

^133^Cs SS MAS NMR experiments were performed at the National High Field NMR facility at the University of Warwick, UK. Spectra were acquired on a Bruker Avance NEO HFXY spectrometer with a MAS III spinning speed controller, using the 4 mm HX (low gamma) H13892 probe. ^133^Cs spectra were acquired at 20.0 T (*B*_0_) with a Larmour frequency of 111.5 MHz, using a rotor synchronised double frequency sweep echo pulse, a measured 0.1 s relaxation delay, spinning at 20.0 MHz, and a total of 86 000 scans.

#### 2.2.4 X-ray absorption spectroscopy (XAS)

XAS was used to measure the strontium and potassium K-edge features (16 keV and 3.6 keV, respectively) and the caesium L-edge features (5 keV) at B18 at Diamond Light Source (DLS) Synchrotron in Harwell, Oxfordshire, UK. Measurements were conducted on the core XAS B18 beamline which covers 2.05–35 keV energy range. The design of B18 has three main optical elements: a collimating mirror, a water-cooled double crystal monochromator, and a focusing mirror. For this study, an unfocused beam was used with a Cr/Pt mirror coating, and a Si111 monochromator. Pellets were produced using finely ground cement powders, combined with polyethylene glycol to give samples with a thickness equal to 2 absorption lengths.

### 2.3 Solution-state characterisation

#### 2.3.1 Inductively coupled plasma – mass spectroscopy (ICP-MS)

Aqueous samples were analysed for Cs and Sr concentrations using ICP-MS (Thermo Fisher iCAP RQ with an ESI PrepFast 4DX autosampler). Concentrations were determined by suitable dilution into 2% (v/v) nitric acid as per standard operating procedures. Calibration curves were prepared by measuring intensities against a series of known calibration standards, which were prepared by diluting single element standard solutions (Fisher Scientific) with 2% (v/v) nitric acid. The ICP data were used to calculate the Cumulative Fraction Leached (CFL) as described in eqn (1).1
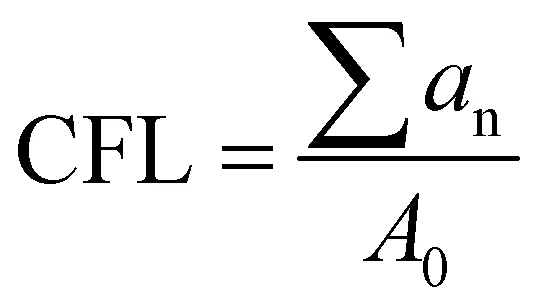
where *a*_n_ is the concentration of radionuclide measured and *A*_0_ is the initial concentration at *t* = 0.

## 3 Mass transport models

In order to determine the mass transfer mechanisms leading to leaching of the caesium and strontium from geopolymers, models must be developed describing the main mass transport processes, including diffusion, dissolution, surface exchange kinetics, and combinations thereof. The mass transfer models are outlined subsequently, as developed from previous literature.^12,15,16^ Using the curve_fit tool from the SciPy package in python, an optimised fit is achieved between the experimental data from the leach tests and the models outlined subsequently. Statistical analysis is performed to understand the goodness of fit of each model and therefore to determine the best-fitting descriptive model of the CFL behaviour.

### 3.1 Diffusive mass transport

It is commonly assumed that, after the initial period of surface wash-off, the leaching behaviour of radionuclides is controlled by diffusion.^22^ To model this, an assumption of diffusion through a semi-infinite cylinder (no axial diffusion due to covering the ends), and therefore the solution of Fick's law in a semi-infinite medium can be applied (2) as follows:2
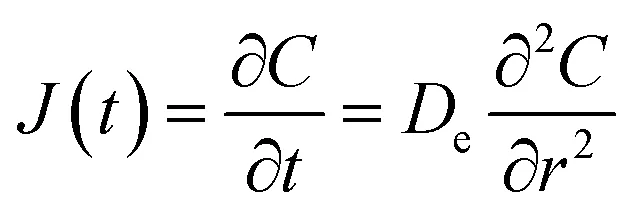
where *C* is the concentration of contaminant (mg m^−3^), *t* is the time (s), *D*_e_ is the effective diffusion coefficient (cm^2^ s^−1^), and *r* is the one-dimensional coordinate/radius (m). For diffusive behaviour, the cumulative fraction leached (CFL) is the flux (*J*(*t*)), and therefore the solution can be written as follows (3):3
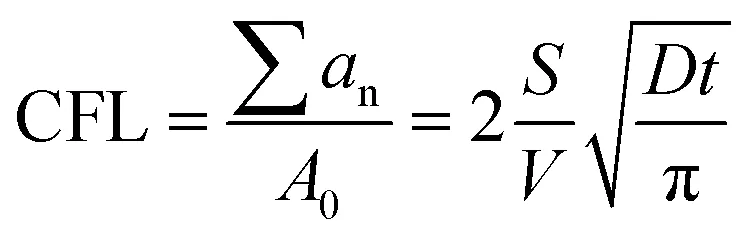


### 3.2 Dissolutive mass transport

As the caesium and strontium can form a structurally significant part of the geopolymer due to the potential ion exchange with potassium during the formation of the geopolymer, the release into solution of these radionuclides can cause a structural change to the matrix. Dissolution behaviour is shown in eqn (4), which assumes a release controlled by the dissolution constant.4
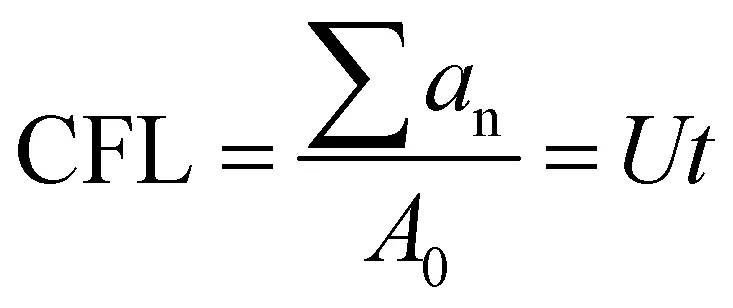
where *U*_0_ is the dissolution constant.

### 3.3 Surface exchange kinetics

Surface phenomena contribute to the mass transport of species from a cementitious or glassy wasteform, described based on the kinetics of exchanges between the surface of a solid and the aqueous solution. The descriptive model for this is shown in eqn (5).5
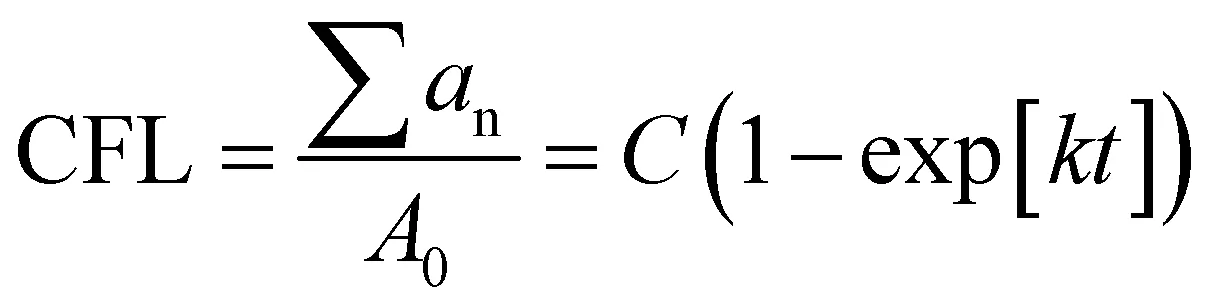
where *C* and *k* are constants based on the initial wash off rate and the exchanges between the surface of the wasteform and the aqueous solution.

### 3.4 Combined mass transport

#### 3.4.1 Diffusion/dissolution model (DDM)

In this model, the mass transfer is assumed to take place due to diffusion through the pores of the geopolymer and through dissolution of the waste matrix, simultaneously. The model is shown in eqn (6).6
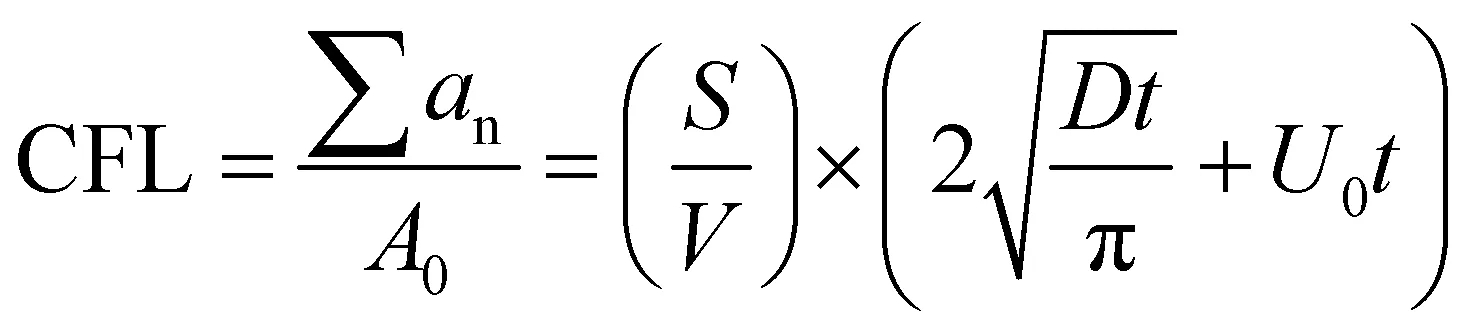


#### 3.4.2 Diffusion/surface exchange kinetics model (DSEM)

In this model, the mass transfer is assumed to take place due to diffusion through the pores and through exchange kinetics between the leachate and the waste matrix on the outer edge. The model is shown in eqn (7).7
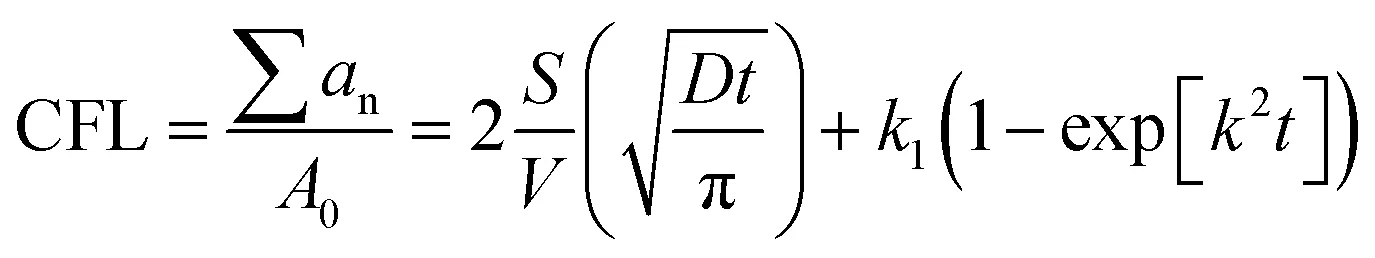


#### 3.4.3 Dissolution/surface exchange kinetics model

In this model, the mass transfer is assumed to occur solely due to dissolution of the waste matrix and exchange between the leachate and the waste matrix. The model is shown in eqn (8).8
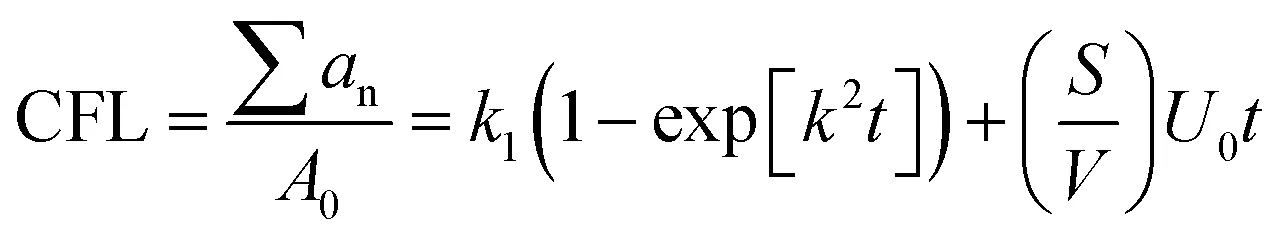


### 3.5 Leachability index (*L*_i_)

In stabilisation/solidification (*S*/*S*) of radioactive and other hazardous wastes, the leachability index (*L*_i_) is an indicative parameter for determining the effectiveness of a waste matrix. *L*_i_ is the logarithm of the effective diffusivity of the radionuclide of interest and a waste form is considered to have acceptable leachability if *L*_i_ is greater than 6.^23^ The leachability index can be calculated with eqn (9).9
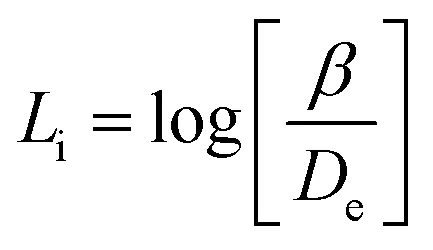
where *β* = 1.0 cm^2^ s^−1^ is a defined constant.

### 3.6 Statistical analysis

#### 3.6.1 *R* ^2^ value

The *R*^2^ value, or the coefficient of determination, is a statistical measure which provides information about the goodness of fit of a model. *R*^2^ is calculated by eqn (10), *i.e.* the sum of the residuals squared divided by the sum of the distance the data is away from the mean all squared, taken from 1. An *R*^2^ of 1 indicates a perfect fit of the model to the data and 0 (or less) indicates no correlation between the model and the data.10
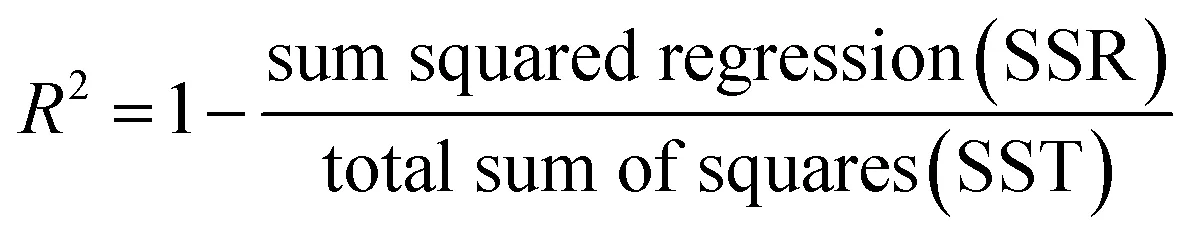


#### 3.6.2 Reduced chi-square value

The reduced chi-square value is used in goodness of fit testing, and indicates whether a data fit has full captured the data, *i.e.* that the extent of the match between observations and estimates is in accord with the error variance. If *χ*_red_^2^ is less than 1, this is considered an over-fit, if *χ*_red_^2^ is greater than 1 then this is considered a “bad” fit, and if *χ*_red_^2^ ≈ 1, then this indicates that the fit has fully captured the data.11
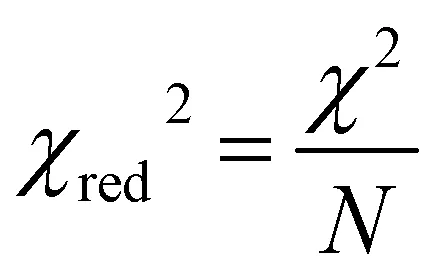
where chi^2^ is the chi-square value, and *N* is the degrees of freedom.12
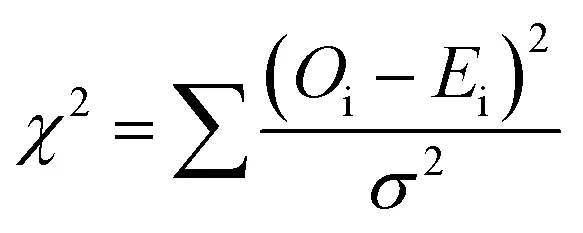
where *O*_i_ is the observed value, *E*_i_ is the expected value, and *σ*^2^ is the variance in the experimental data.

## 4 Results and discussion

### 4.1 Cumulative fraction leached

**Fig. 1:**
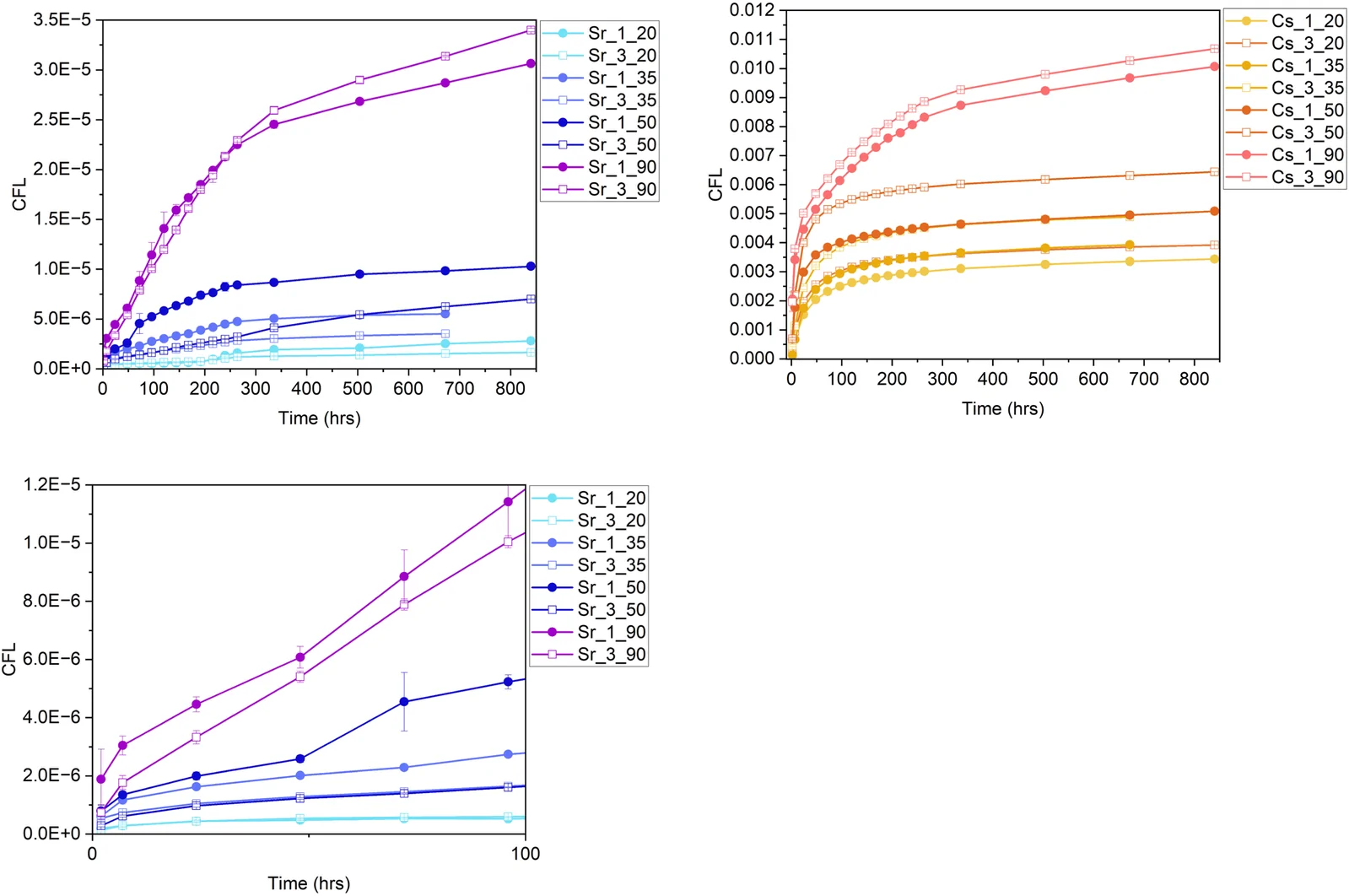
CFL of Sr (left) and Cs (right) from metakaolin-based geopolymer monoliths containing 1 or 3 wt% Sr or Cs during ASTM C1308 monolithic leach testing at 35, 50, and 90 °C. Symbols represent the mean of triplicate measurements. The first 100 hours of Sr release is shown below.

To evaluate the performance of the geopolymer wasteforms in different temperatures, the cumulative fraction leached (CFL) plots for 35, 50, and 90 °C are shown in Fig. 1, alongside the first 100 hours of the leach test for Sr. The CFL for 20 °C is also included to allow for an intuitive comparison across the full temperature range (20–90 °C).^17^ At the discrete time points, leachate aliquots were collected and analysed by ICP-MS to quantify the experimental release of Cs and Sr from the waste matrix. The CFL, defined as the ratio of the cumulative mass released to the initial mass in the matrix, was calculated to assess the experimental release. All leaching experiments were performed in triplicate and following the ASTM C1308 standard, ensuring low experimental error.

Overall, the plots reveal a significantly larger CFL for Cs compared with Sr across all temperatures. This is mainly dependent on the incorporation of the cations into the geopolymer. Sr tends to precipitate as water insoluble SrCO_3_ with some partially Sr-substituted cation binding sites within the gel, whereas Cs tends to sit in the cation binding sites.^17^

Furthermore, there is a pronounced temperature dependency for the CFL for both Cs and Sr. For Sr loaded at 1 and 3%, the maximum values increased from 5.5 × 10^−6^ and 3.5 × 10^−6^ to 1.0 × 10^−5^ and 7.0 × 10^−6^, and to 3.1 × 10^−5^ and 3.4 × 10^−5^, for 35, 50, and 90 °C, respectively. This represents an increase of almost an order of magnitude over the temperature range. Similarly, for Cs at 1 and 3%, the maximum CFL values increased from 0.0039 and 0.0049 at 35 °C to 0.0051 and 0.0064 at 50 °C, to 0.010 and 0.011 at 90 °C, indicating nearly a tripling of the release fraction.

The increase in CFL could be attributed to structural changes within the geopolymer due to the temperature increases such as the increase in solubility of SrCO_3_ with temperature, from 11 mg L^−1^ at 18 °C to 650 mg L^−1^ at 100 °C,^24^ or due to changes in the mass transport kinetics, such as increased diffusion rates. Therefore, both structural characterisation and mass transport analysis are presented in this work.

Finally, the CFL release curves for the Sr-containing samples do not clearly show the same staggered release behaviour observed in the control samples at 20 °C.^17^ One possible explanation is that the local crystallisation of K–A–S–H into a zeolitic phase does not occur at higher temperatures. The CFL during the first 100 hours of the test provides some evidence of a smaller release event occurring in the 50 and 90 °C samples at shorter timescales. This suggests that a local zeolitic phase may still be forming but at a faster rate under higher temperature conditions. Previous studies have shown that curing geopolymers at higher temperatures can promote crystallisation of the amorphous matrix into a zeolite.^25,26^ Therefore, it is likely that local crystallisation is still occurring, but proceeds faster than at 20 °C. This, combined with the increase in other mass transport processes such as diffusion, which generally increases with temperature, and surface wash off, which dominates at early timescales, likely contributes to the less pronounced staggered release behaviour compared with the control samples.

To assess whether the leaching behaviour of the matrix elements such as Si and Al is congruent with that of Cs and Sr, the leaching profiles for Al, Si, and K are included in the SI. This revealed the exceptional structural stability of the geopolymers under the conditions tested. The Al and Si release profiles were markedly similar, and exhibited very low release rates, suggesting minimal dissolution of the geopolymer framework itself as these elements constitute its primary structure. There is a slight temperature dependency, suggesting dissolution of the K–A–S–H gel may increase with temperature, although the release rates are minimal.

The CFL can be used to calculate the effective diffusivity constant from the slope of the straight line plot of CFL *vs. t*^1/2^ using eqn (13). From this, the leachability indices (*L*_i_) can be determined and are displayed in Table 2. Under all conditions tested, the *L*_i_ value exceeded the industry minimum accepted value of 6, within the error calculated. This indicates the high resistance of the geopolymer wasteforms to leaching under the temperatures tested and generally exceeds *L*_i_ values demonstrated by other cementitious systems.^27,28^13
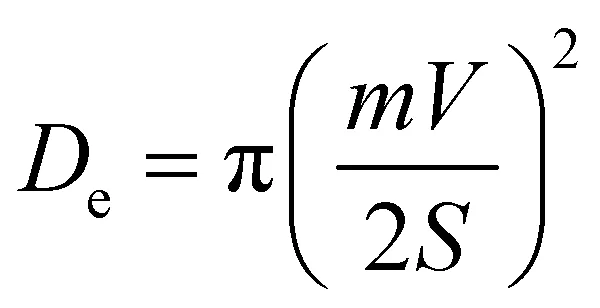
where *V* is the volume of the sample (cm^3^) and *S* is the effective surface area of the sample (cm^2^).

**Table 2:** Calculated diffusion coefficients and associated leachability indices for Sr and Cs

Sample	*D*_e_ (cm^2^ s^−1^)	*L*_i_
Sr_1_35	1.6 × 10^−18^ ± 6.0 × 10^−23^	17.8 ± 0.00002
Sr_3_35	3.9 × 10^−19^ ± 7.2 × 10^−24^	18.4 ± 0.000008
Cs_1_35	1.0 × 10^−13^ ± 2.7 × 10^−18^	13.0 ± 0.00001
Cs_3_35	1.3 × 10^−13^ ± 1.2 × 10^−18^	12.9 ± 0.000004
Sr_1_50	1.8 × 10^−18^ ± 1.2 × 10^−22^	17.7 ± 0.00003
Sr_3_50	2.1 × 10^−18^ ± 5.6 × 10^−24^	17.7 ± 0.000001
Cs_1_50	6.6 × 10^−14^ ± 4.2 × 10^−19^	13.2 ± 0.000003
Cs_3_50	5.1 × 10^−14^ ± 1.1 × 10^−19^	13.3 ± 0.000001
Sr_1_90	2.9 × 10^−17^ ± 1.8 × 10^−21^	16.5 ± 0.00003
Sr_3_90	4.4 × 10^−17^ ± 6.2 × 10^−22^	16.4 ± 0.000006
Cs_1_90	9.8 × 10^−13^ ± 1.3 × 10^−17^	12.0 ± 0.000006
Cs_3_90	1.4 × 10^−12^ ± 1.1× 10^−20^	11.9 ± 0.000002

### 4.2 X-ray diffraction

**Fig. 2:**
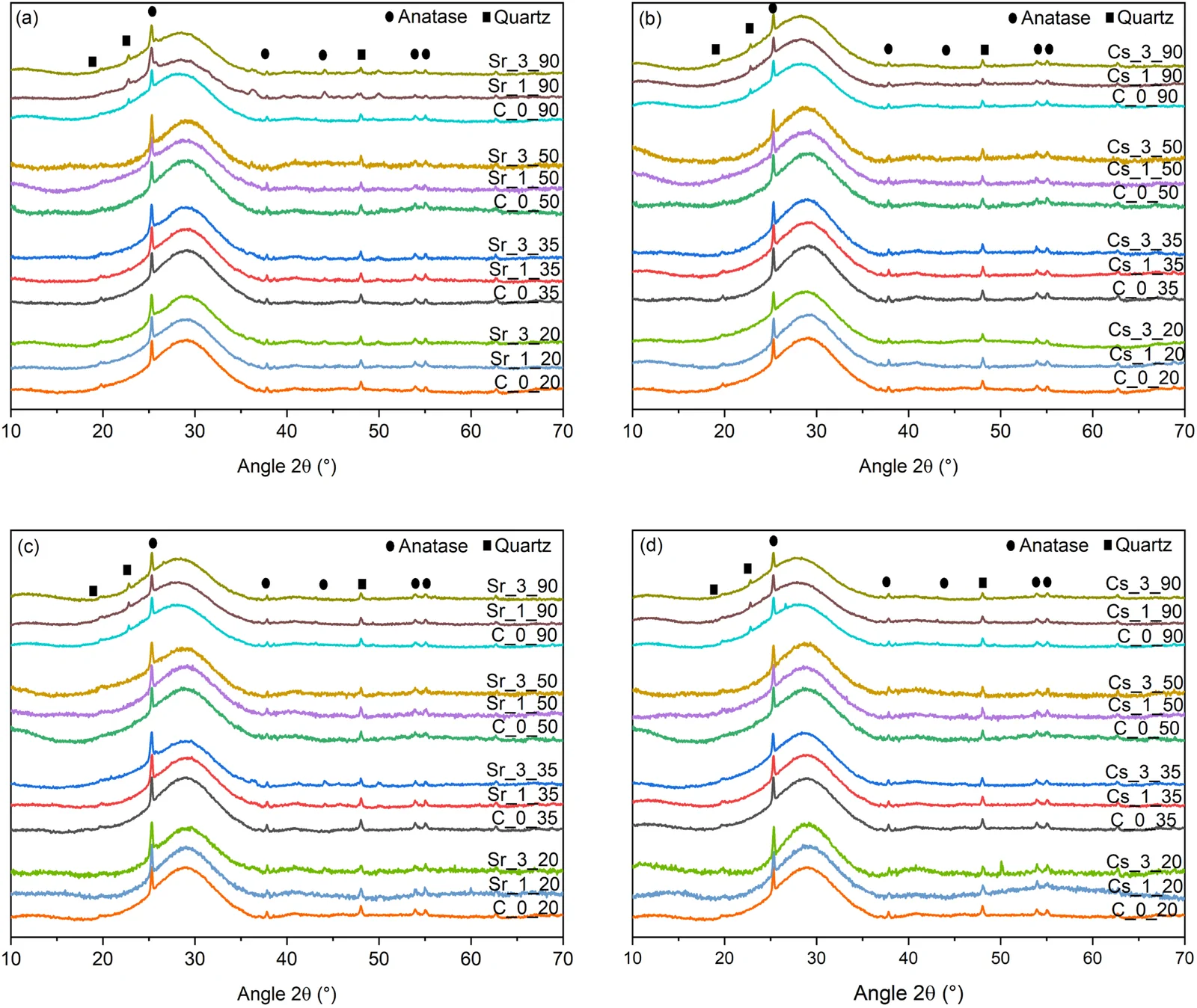
Powder XRD patterns of geopolymer samples before leaching for (a) Sr and (b) Cs samples, and after ASTM C1308 leach testing at 35, 50, and 90 °C for (c) Sr and (d) Cs samples. Sample codes are formatted as RN–X–T, where RN denotes the incorporated radionuclide analogue (Sr or Cs), *X* is the loading (wt%), and *T* is the leaching temperature. Quartz and anatase reflections originate from the metakaolin precursor.

X-ray diffraction data shown in Fig. 2 are consistent with the formation of an aluminosilicate gel, with a broad, amorphous feature at 29° 2*θ*, indicating the formation of a K–A–S–H gel. Furthermore, there are small crystalline impurities, mostly attributed to the presence of quartz and anatase impurities in the metakaolin precursor or in sand due to preparation of the samples. The post-leach samples remain essentially unchanged within the detection limits of laboratory XRD. No new crystalline phases were detected and the amorphous K–A–S–H hump remained unchanged, indicating that elevated leaching temperatures do not induce significant bulk crystallisation or degradation of the geopolymer framework. Minor local structural rearrangements or secondary phases present below the XRD detection limit (typically 1 wt%) cannot, however, be excluded.

### 4.3 Fourier transform infrared spectroscopy

**Fig. 3:**
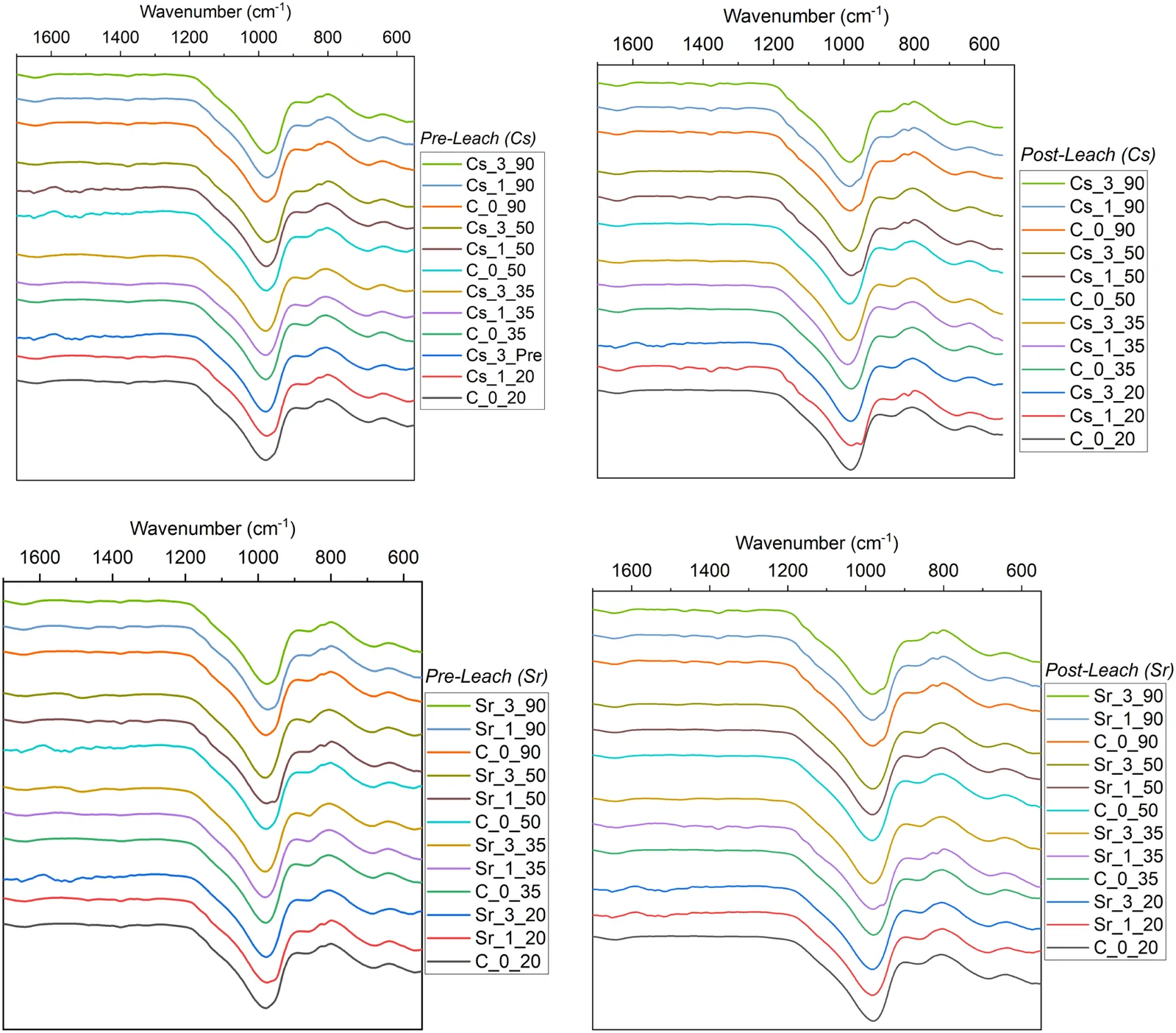
ATR-FTIR spectra of geopolymer samples before leaching and after ASTM C1308 leach testing at 35, 50, and 90 °C, showing the stability of the aluminosilicate (K–A–S–H) framework following exposure to elevated temperatures.

The FTIR spectra, shown in Fig. 3, are consistent with the formation of a geopolymer gel. The large band at 950–1200 cm^−1^ is indicative of the asymmetric stretching of a T–O–T bond (where T is Al or Si), characteristic of a K–A–S–H structure.^29^ The fingerprint region at lower wavenumbers contains a number of bands indicative of a geopolymer; symmetric stretching of Al–O–Si (560 cm^−1^),^30^ the formation of a potassium substituted zeolite A, K-LTA (673 cm^−1^),^31^ symmetric stretching of T–O–T,^6^ and the asymmetric stretching of T–O–T bonds linking AlO_4_ and SiO_4_ tetrahedral bonds,^2^ respectively. There is the indication of some carbonation, as indicated by the small bands at 1300, 1370, and 1470 cm^−1^, consistent with formation of carbonate ions. This is to be expected as the samples were not handled in an anoxic atmosphere. It is clear that the pre- and post-leach samples are nearly identical, with minimal shifts in bands and a similar degree of carbonation, likely contributing to Sr retention through the formation of insoluble SrCO_3_ precipitates within the pores. Overall, there is little to no change in the bonds present in the samples regardless of leaching temperature.

### 4.4 Solid state nuclear magnetic resonance spectroscopy

**Fig. 4:**
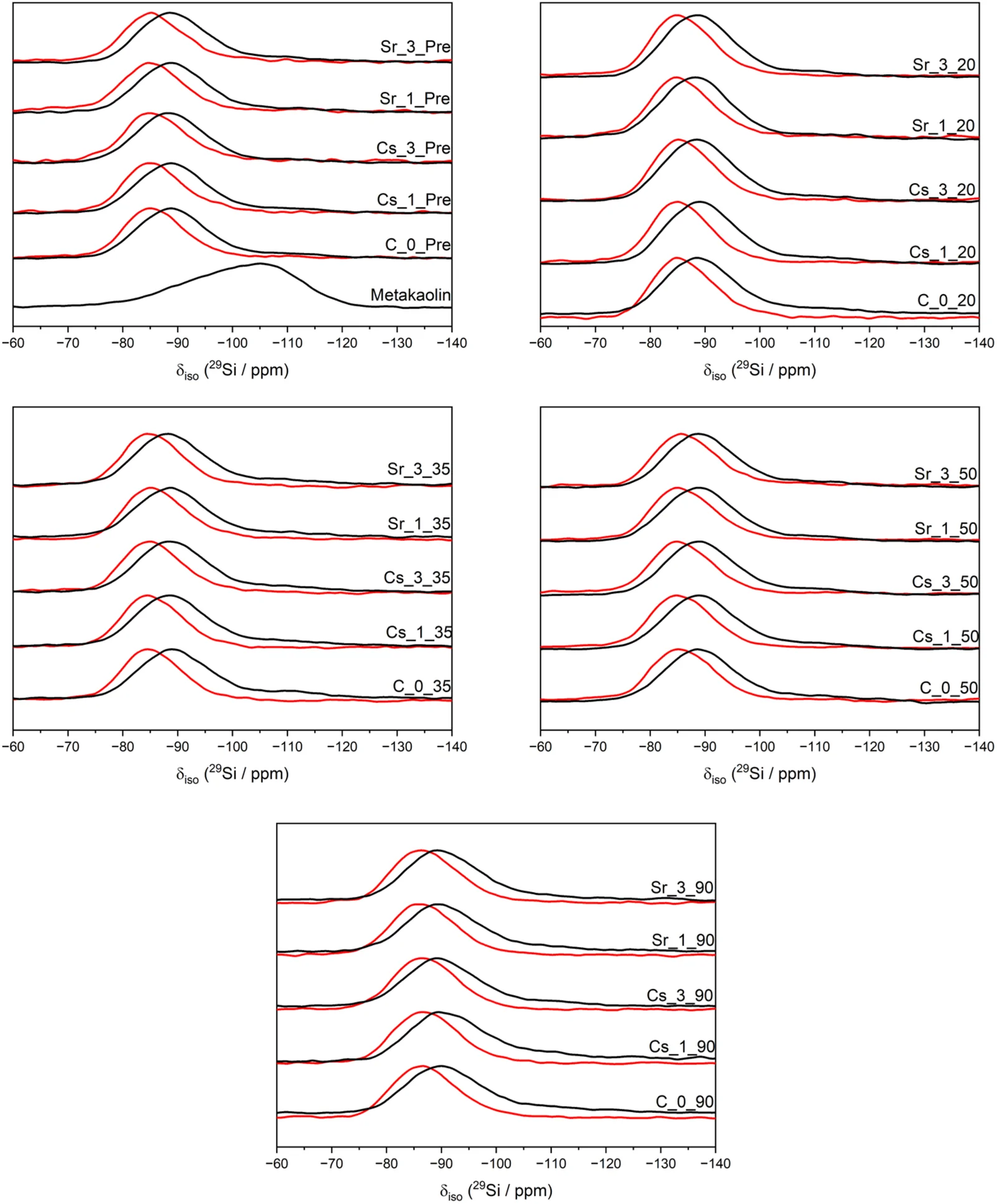
^29^Si MAS (*B*_0_ = 11.7 T, *ν*_R_ = 12.5 kHz, shown in black) NMR and ^1^H–^29^Si CPMAS (*B*_0_ = 11.7 T, *ν*_R_ = 12.5 kHz and Hartmann–Hahn contact period *t* = 1.7 ms, shown in red) NMR spectra, showing local silicon environments within the K–A–S–H gel.

The ^29^Si MAS NMR and ^1–29^Si MAS NMR spectra are shown in Fig. 4. The ^29^Si MAS NMR spectra are centred around −85.5 ppm for the pre-leach samples, with some small shifts after leaching. The samples leached at 35, 50, and 90 °C shift by a maximum of 0.2, 0.8, and 0.9 ppm, respectively. This is within the error of the instrument and therefore leaching does not have a significant effect on the bonding environments within the geopolymer. The broad resonances show a consistent lineshape and span from *δ*_iso_ = −75 ppm to −110 ppm, indicating an extensive distribution of Q^4^(*m*Al) environments (where 0 ≤ *m* ≤ 4)^2^^29^Si environments within the geopolymer. Si sites are generally identified using notation of the type Q^*n*^(*m*Al) with 0 ≤ *m* ≤ *n* ≤ 4, where Si in tetrahedral coordination (represented by Q) is bonded to *n* other tetrahedral atoms (*m* of which are Al) *via* oxygen bridges.^18^

The ^1^H–^29^Si cross-polarisation MAS NMR spectra are assessed to find the sites in the hydrated K–A–S–H gel as it favours the sites near to a proton (*i.e.* the hydrogen in water). Therefore, these favour higher wavenumbers as they do not include the unreacted precursor in the geopolymer. Deconvolution of the ^1^H–^29^Si CP MAS spectra reveal four distinct silicon sites within the K–A–S–H gel at *δ*_iso_ values of −86 ppm, −90 ppm, −95 ppm, and −102 ppm ± 5 ppm, corresponding to Q^4^(4Al), Q^4^(3Al), Q^4^(2Al), and Q^4^(1Al) sites. Deconvolution of the ^29^Si MAS NMR spectra reveal a fifth silicon site (Q^4^(0Al)), identified as the silicon sites within the unreacted metakaolin precursor. It also allows quantification of the sites identified from the ^1^H–^29^Si CP MAS spectra, which preferentially identified silicon sites with higher aluminium substitution as they are closer to protons. The ^29^Si MAS NMR spectra allows quantification of the sites present from the relative integral areas and the corresponding Si/Al ratio, as calculated from Engelhardt's formula (eqn (14)). The relative integral areas and the Si/Al ratios are displayed in Table 3. Deconvolutions for 20 °C are included in the SI.14
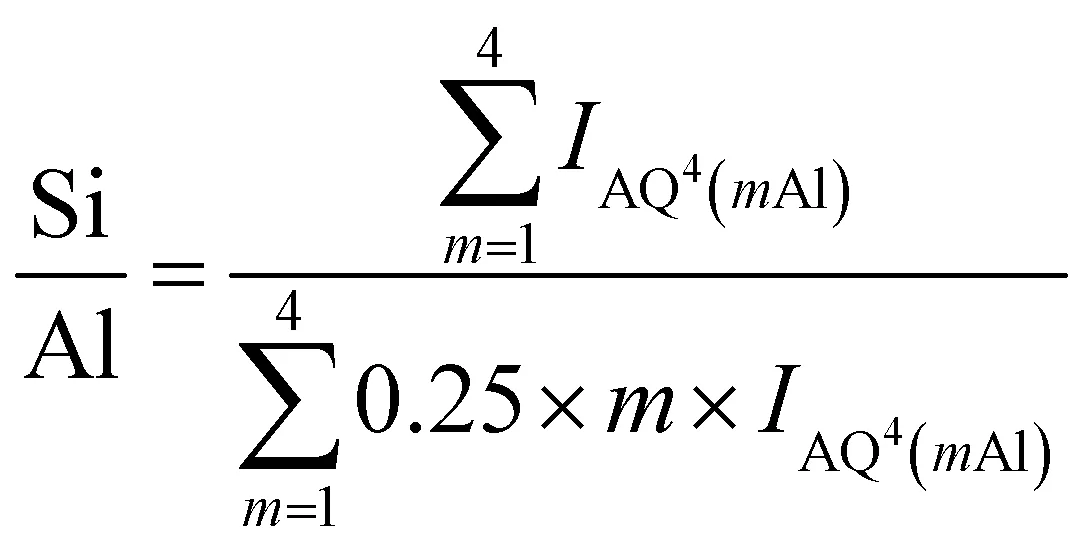
where *I*_AQ^4^(*m*Al)_ refers to the normalised relative integral areas of the resonances in the ^29^Si MAS NMR deconvolutions of each silicon site in the geopolymer gel.

**Table 3:** Relative integral areas for silicon environments within K–A–S–H gel in each sample and associated Si/Al ratios

Site	Q^4^(4Al)	Q^4^(3Al)	Q^4^(2Al)	Q^4^(1Al)	Si/Al
C_0_Pre	23.9	38.6	32.1	5.4	1.42
Sr_1_Pre	18.2	44.3	31.8	5.7	1.45
Sr_3_Pre	17.3	47.4	33.1	2.3	1.43
Cs_1_Pre	21.3	36.1	37.7	4.9	1.46
Cs_3_Pre	26.5	38.1	29.9	5.4	1.40
C_0_35	18.0	44.3	35.3	2.4	1.44
Sr_1_35	24.4	39.0	34.1	2.4	1.40
Sr_3_35	25.4	42.9	26.2	5.6	1.39
Cs_1_35	23.1	38.5	33.1	5.4	1.43
Cs_3_35	24.2	37.9	23.3	5.6	1.43
C_0_50	26.8	40.2	31.2	1.8	1.37
Sr_1_50	27.0	33.6	35.2	4.1	1.41
Sr_3_50	24.8	43.8	25.6	5.8	1.39
Cs_1_50	22.2	43.1	31.1	3.6	1.41
Cs_3_50	23.3	38.8	34.5	3.4	1.42
C_0_50	23.5	43.1	22.5	10.8	1.43
Sr_1_90	19.4	47.8	26.7	6.1	1.43
Sr_3_90	23.1	44.4	28.7	3.7	1.39
Cs_1_90	17.3	40.7	29.6	12.3	1.52
Cs_3_90	22.2	43.4	24.2	10.1	1.44

**Fig. 5:**
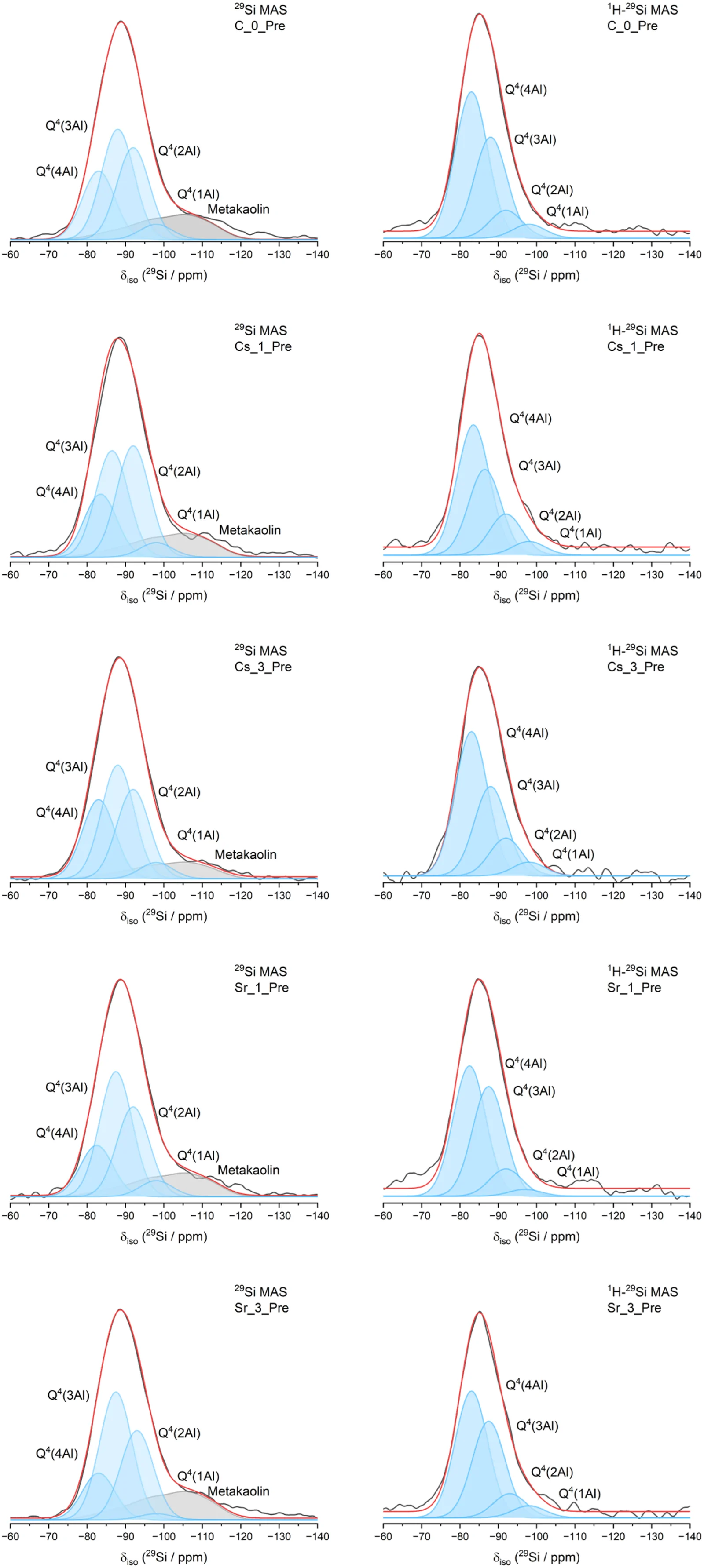
^29^Si MAS (*B*_0_ = 11.7 T, *ν*_R_ = 12.5 kHz) and ^1^H–^29^Si CPMAS (*B*_0_ = 11.7 T, *ν*_R_ = 12.5 kHz and Hartmann–Hahn contact period *t* = 1.7 ms) NMR spectra and associated deconvolutions for pre-leach geopolymer gels. The data are shown in black, the fit (the sum of the deconvoluted peaks) is shown in red, the peaks attributed to Si sites are shown in blue, and peaks attributed to Si sites in unreacted metakaolin in grey.

**Fig. 6:**
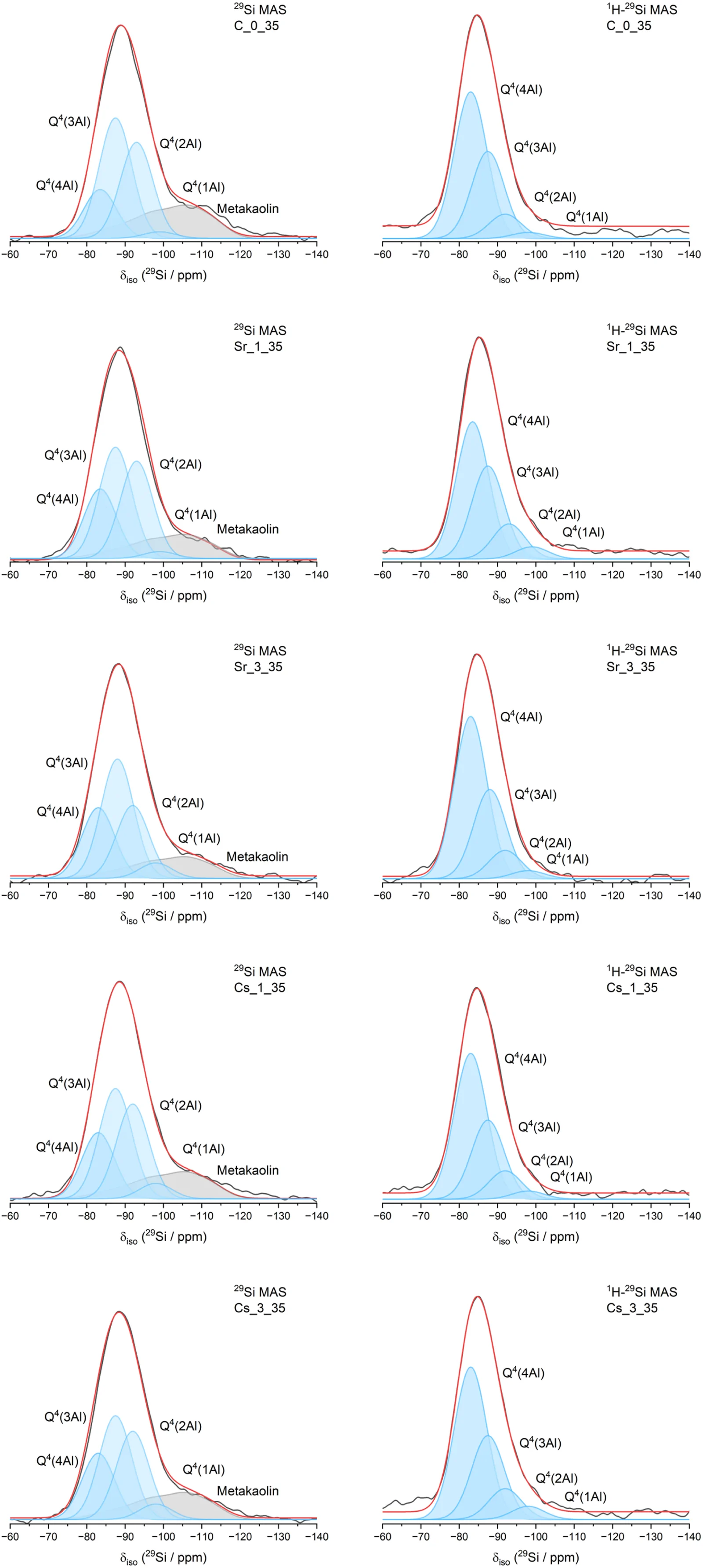
^29^Si MAS (*B*_0_ = 11.7 T, *ν*_R_ = 12.5 kHz) and ^1^H–^29^Si CPMAS (*B*_0_ = 11.7 T, *ν*_R_ = 12.5 kHz and Hartmann–Hahn contact period *t* = 1.7 ms) NMR spectra and associated deconvolutions for geopolymer gels leached at 35 °C. The data are shown in black, the fit (the sum of the deconvoluted peaks) is shown in red, the peaks attributed to Si sites are shown in blue, and peaks attributed to Si sites in unreacted metakaolin in grey.

**Fig. 7:**
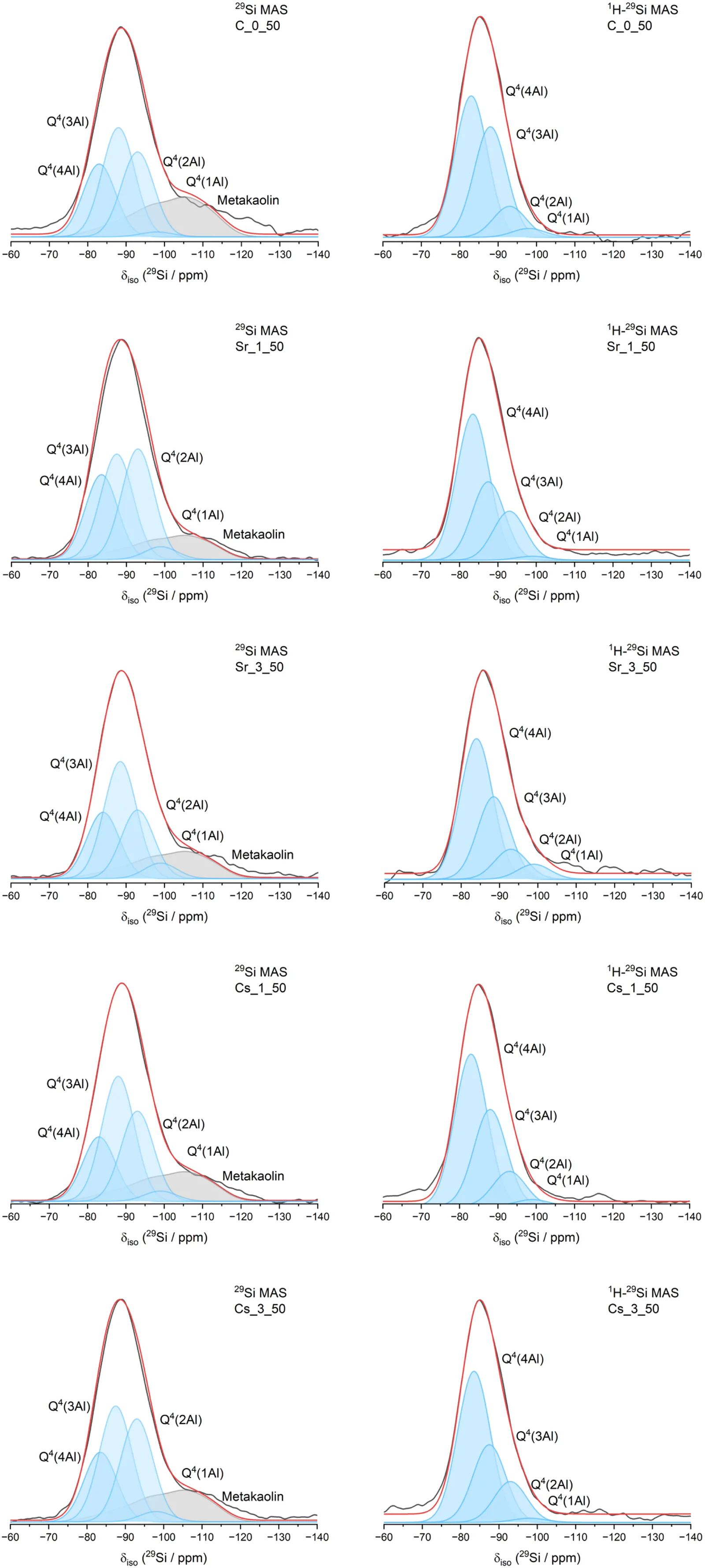
^29^Si MAS (*B*_0_ = 11.7 T, *ν*_R_ = 12.5 kHz) and ^1^H–^29^Si CPMAS (*B*_0_ = 11.7 T, *ν*_R_ = 12.5 kHz and Hartmann–Hahn contact period *t* = 1.7 ms) NMR spectra and associated deconvolutions for geopolymer gels leached at 50 °C. The data are shown in black, the fit (the sum of the deconvoluted peaks) is shown in red, the peaks attributed to Si sites are shown in blue, and peaks attributed to Si sites in unreacted metakaolin in grey.

**Fig. 8:**
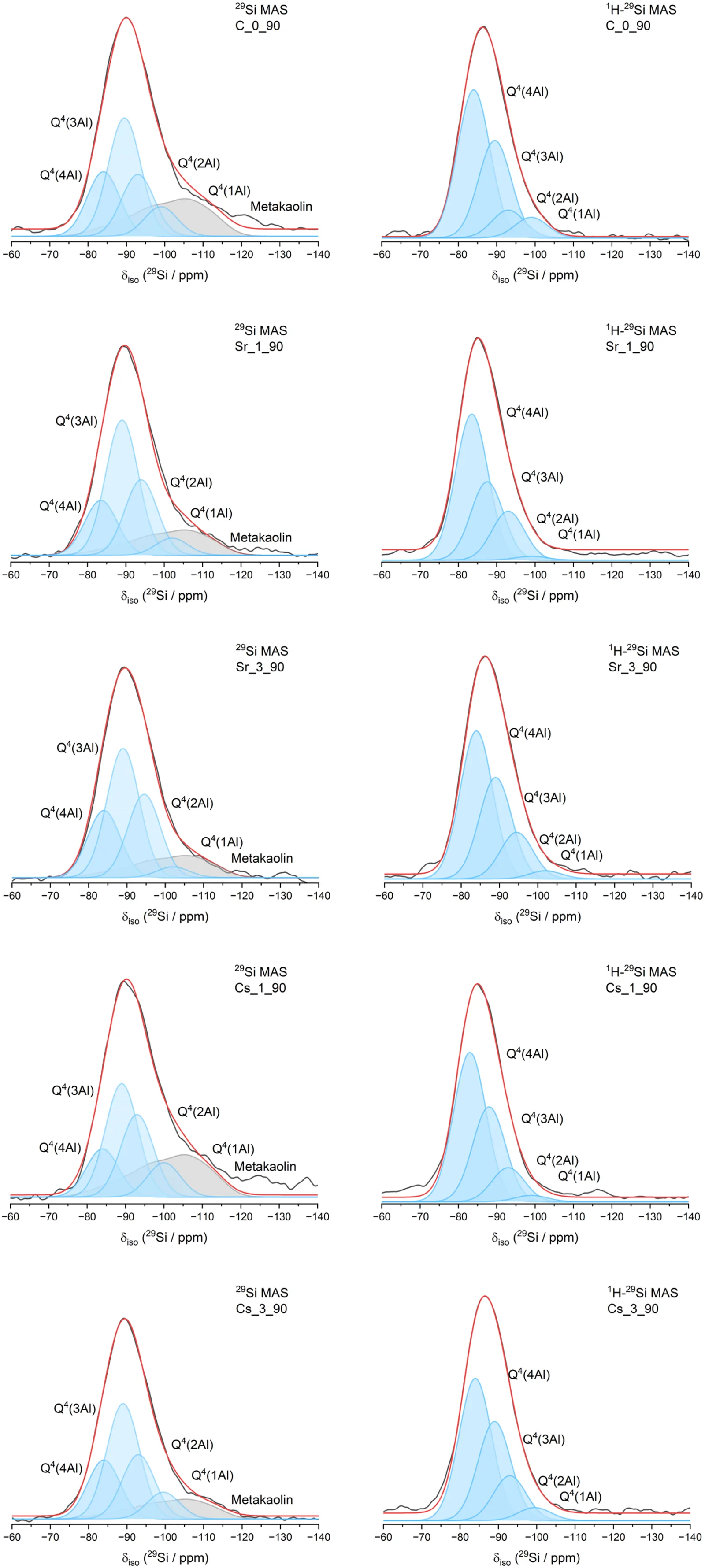
^29^Si MAS (*B*_0_ = 11.7 T, *ν*_R_ = 12.5 kHz) and ^1^H–^29^Si CPMAS (*B*_0_ = 11.7 T, *ν*_R_ = 12.5 kHz and Hartmann–Hahn contact period *t* = 1.7 ms) NMR spectra and associated deconvolutions for geopolymer gels leached at 90 °C. The data are shown in black, the fit (the sum of the deconvoluted peaks) is shown in red, the peaks attributed to Si sites are shown in blue, and peaks attributed to Si sites in unreacted metakaolin in grey.

This deconvolution allows trends in the ratios to be observed. With increasing temperature, the average Si/Al ratio by looking across the whole dataset decreases until 50 °C, but then increases again at 90 °C to a similar value to that seen in the pre-leach samples. However, these averages are very similar, with average Si/Al for pre-leach, 35 °C, 50 °C, and 90 °C of 1.43, 1.42, 1.40, and 1.44, which could be within the error of the instrument. It is also clear that in general the caesium samples have higher Si/Al ratios when compared with the control or strontium samples. The reasons for this are twofold; the presence of caesium and strontium tends to increase the amount of Q^4^(3Al) and caesium-containing samples show a lower Q^4^(4Al) percentage overall. Therefore, this gives rise to the higher Si/Al values. These slight shifts in the percentage ratio are due to the relative comparative size of caesium and strontium compared with potassium, which is the original charge-balancing cation and has a much smaller atomic radius. Overall, the presence of caesium and strontium in the geopolymer and leaching at higher temperatures does not have a particularly significant effect on the Si/Al ratios or on the local structure around the Si atoms (Fig. 5–8).

**Fig. 9:**
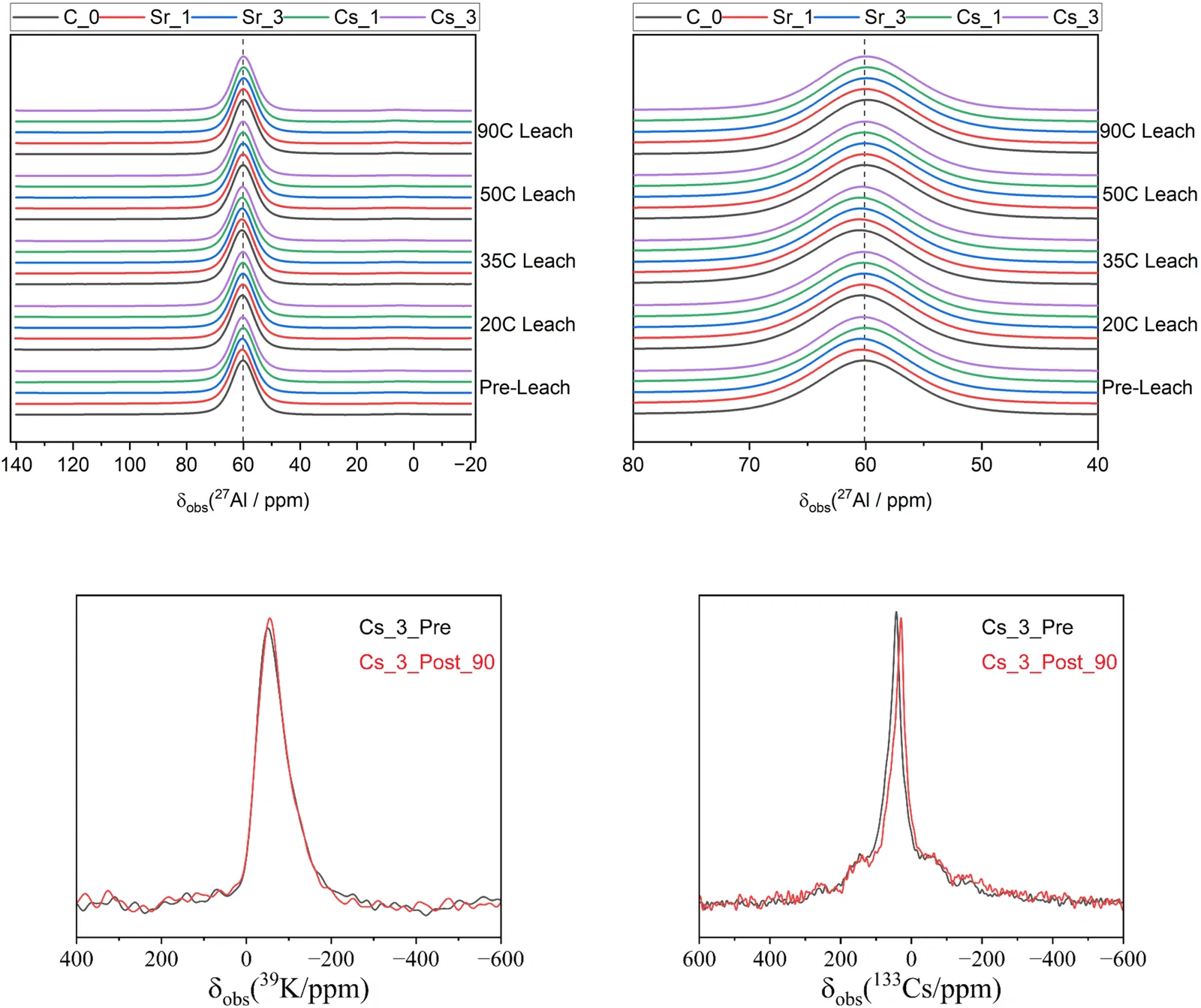
(Top) ^27^Al MAS NMR spectra (*B*_0_ = 11.7 T, *ν*^R^ = 12.5 kHz) for each geopolymer gel. (Bottom) ^39^K MAS NMR spectra (*B*_0_ = 11.7 T, *ν*^R^ = 12.5 kHz) for the Cs_3 geopolymer gel, pre- and post-leaching at 90 °C and ^133^Cs MAS NMR spectra (*B*_0_ = 11.7 T, *ν*^R^ = 12.5 kHz) for the Cs_3 geopolymer gel, pre- and post-leaching at 90 °C.

The ^27^Al MAS NMR data are plotted in Fig. 9. The expected resonances will be seen at *δ*_obs_ = 56, 33, and 8 ppm,^18^ attributed to tetrahedral, pentahedral, and octahedral coordination. In these samples, there are very minor resonances observed at chemical shift *δ* = 8 ppm, but the main resonance band is seen at *δ*_obs_ = 60 ppm, attributed to a AlO_4_ structure within the K–A–S–H gel. This is expected when aluminium is in a fully polymerised tetrahedral site, as seen in a K–A–S–H gel^2^ due to the excess of aluminium ions within the cement. All samples display a very consistent lineshape with only small shifts in the centre of the resonance bands (50.9 ppm ≤ *δ*_obs_ ≤ 60.4 ppm), indicating little to no variation in the aluminium sites. There is a very slight shift towards higher chemical shift values in the pre-leach strontium samples due to slight shielding of the ^27^Al nucleus from the divalent Sr^2+^ ion,^2^ but this is not observed in the post-leach samples, indicating some structural reordering of the aluminium sites in the strontium-containing samples as a result of the leaching, but this is very minimal (≈0.3 ppm).

High field ^39^K MAS NMR data were obtained for the Cs_3 samples before and after leaching and are also shown in Fig. 9. The data exhibit a broad resonance spanning from *δ*_obs_ = 20 ppm to −300 ppm, centred at *δ*_obs_ = −53 ppm. This indicates charge-balancing extra-framework K^+^ ions within a (N, K)–A–S–H-type gel. The width and lineshape of the chemical shift distribution in the ^39^K MAS NMR data is very similar for the sample before and after leaching at both 90 °C and 20 °C (data published previously in ref. 17), indicating no significant changes to the local environment of K as a result of leaching.

The ^133^Cs MAS NMR data for the pre-leach sample has been published previously^17^ and is included here for comparison with ^133^Cs MAS NMR data for the sample leached at 90 °C (see Fig. 9). The data for both the pre-leached and post-leached (90 °C) sample exhibits a generally symmetrical line-shape with a sharp peak and a broad base and is indicative of a Cs ion in a structure similar to that seen in zeolite-A, with a chemical shift *δ*_obs_ = 42.7 ppm. This indicates that the Cs is bound into the charge-balancing sites of the aluminosilicate gel, in place of the K^+^ ions in the K–A–S–H gel. However, there is a significant shift towards lower *δ* values after leaching, with a chemical shift *δ*_obs_ = 29 ppm. This is more indicative of Cs in a zeolite-X structure, which has a different shape to zeolite-A and offers larger pore sizes. Furthermore, the peak becomes narrower after leaching, indicating that the Cs environment is more ordered or symmetric, possibly due to leaching of some of the loosely bound Cs causing some structural rearrangement. Similar changes were observed in the data for the sample leached at 20 °C,^17^ however in that study when samples were leached at 20 °C the ^133^Cs MAS NMR data exhibited a chemical shift of *δ* = 35 ppm, indicating that leaching at 90 °C results in additional shielding of the ^133^Cs compared to leaching at 20 °C.

### 4.5 X-ray absorption spectroscopy

#### 4.5.1 X-ray absorption near-edge structure (XANES) spectroscopy

**Fig. 10:**
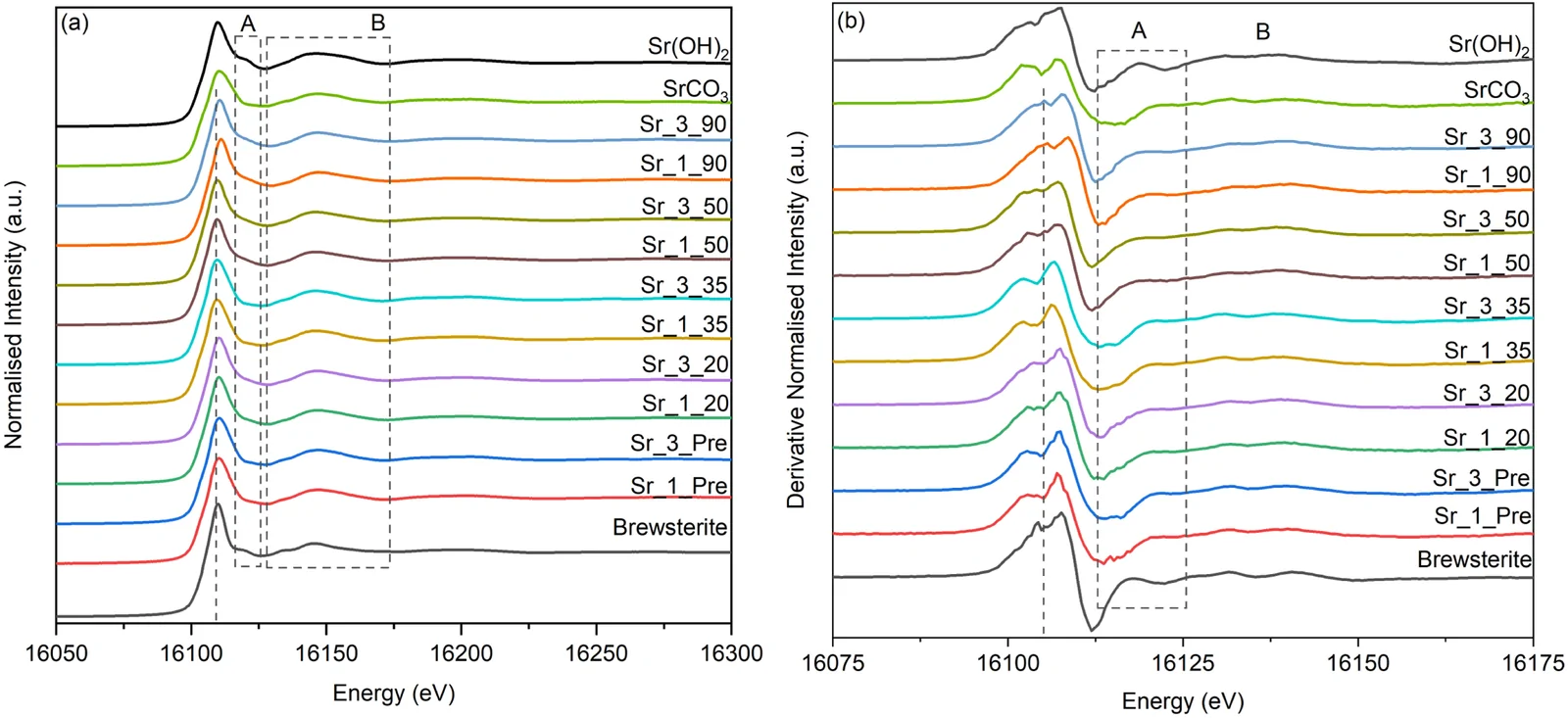
Sr K-edge XANES spectra of Sr-containing geopolymer samples before leaching and after leaching at 35, 50, and 90 °C. (a) Normalised absorption spectra. (b) First derivatives of the spectra. Reference compounds (brewsterite-Sr, SrCO_3_ and Sr(OH)_2_) are included for comparison of local Sr coordination environments.

XANES is a diagnostic tool used to probe the local structure of an element in a sample, in this case strontium is probed. Data was unable to be obtained for caesium due to an edge overlap with titanium (an impurity in metakaolin) at the energy required, and is therefore omitted from this work. Fig. 10 displays the data for the pre-leach samples, and then the samples leached at temperature. Previous work has shown that strontium has a similar local coordination to that seen in SrCO_3_, Sr(OH)_2_, or brewsterite-Sr (a zeolitic mineral containing strontium) and these are overlaid for structural comparison.^18–20^

The strontium K-edge is characterised by a smoothly rising absorption edge into a single peak at the maximum. There are very slight shifts in the maxima (a guide line is included to draw the eye), as elucidated by Fig. 10b, the first derivative of the peaks which can draw out some features. Although the rising edge appears relatively featureless in Fig. 10a, the peak is actually split into a doublet. The brewsterite doublet has two sharp peaks with a relatively low intensity, whereas the SrCO_3_ peaks are broad with a low intensity. Finally, the Sr(OH)_2_ derivative reveals a broad, low intensity peak and a broad, high intensity peak. When compared to the spectra for the samples, it is clear there is some structural reordering during leaching. The doublet for the pre-leach samples looks like that of brewsterite, whereas the post-leach samples look increasingly more like Sr(OH)_2_ or SrCO_3_ with increasing temperature.

The feature on the shoulder of the main peak (feature A) is visible on the brewsterite and Sr(OH)_2_ peaks, but is not clearly identifiable on the SrCO_3_ spectrum on Fig. 10a. The first derivative displays this more clearly, with a clear, broad peak labelled feature A. This feature is not present on any of the samples, which resemble the feature as seen on the SrCO_3_ spectrum. Finally, feature B is a broad peak, which is more intense for Sr(OH)_2_ and has a slight shoulder for the brewsterite. However, the derivative plot reveals that the samples mostly resemble SrCO_3_ again in this region.

Overall, the XANES spectra indicate a mixture of local structures similar to those in brewsterite-Sr, SrCO_3_, and Sr(OH)_2_ across the samples, with a tendency to experience some structural reordering after leaching due to a loss of strontium.

### 4.6 Leaching characteristics

#### 4.6.1 Single source mass transport models

**Fig. 11:**
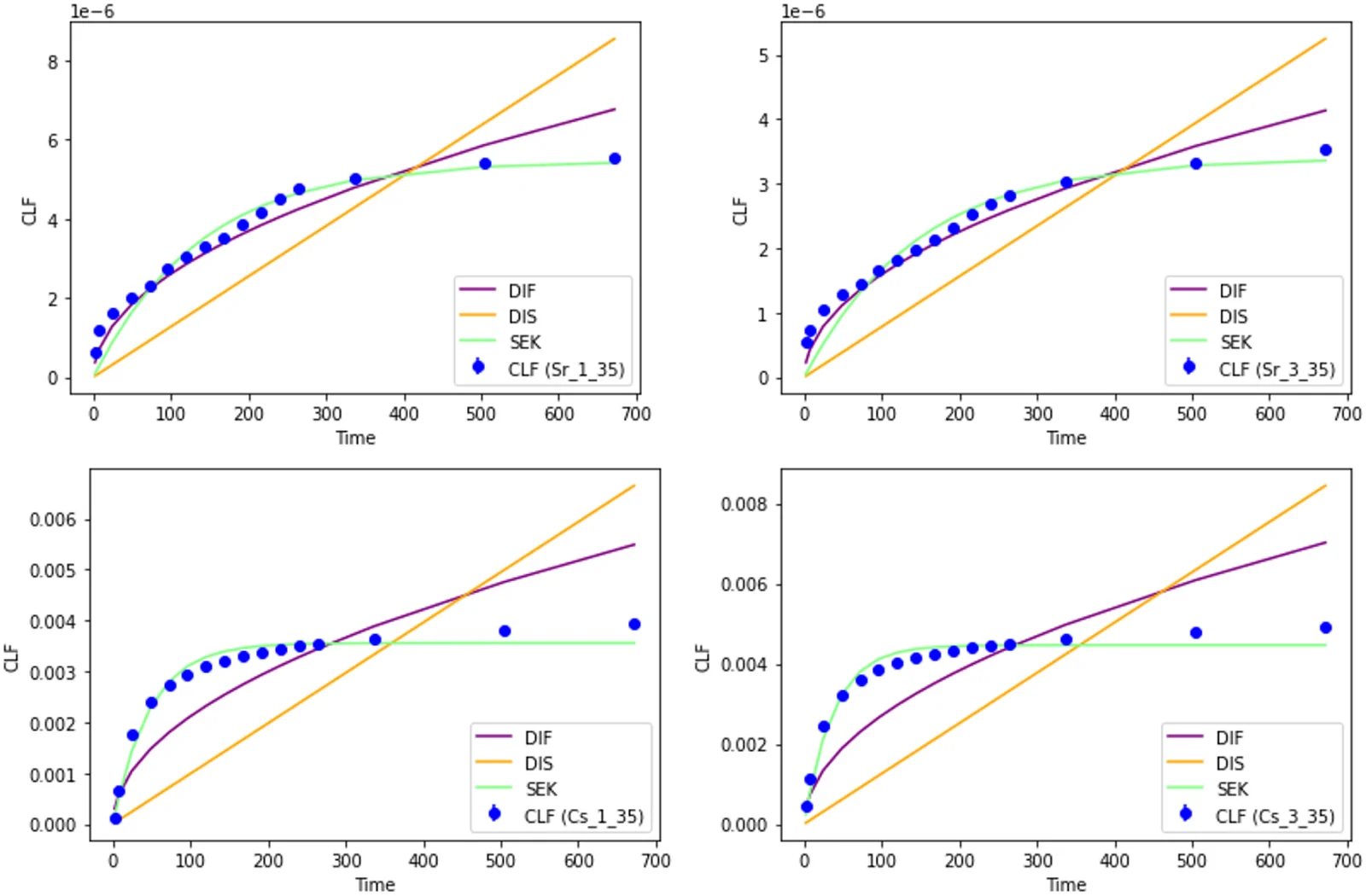
Fits of the single-mechanism mass transport models (diffusion, dissolution and surface exchange kinetics) to the experimental CFL data for Sr (top) and Cs (bottom) during leaching at 35 °C. Symbols denote experimental measurements and solid lines represent model predictions obtained by nonlinear regression.

**Fig. 12:**
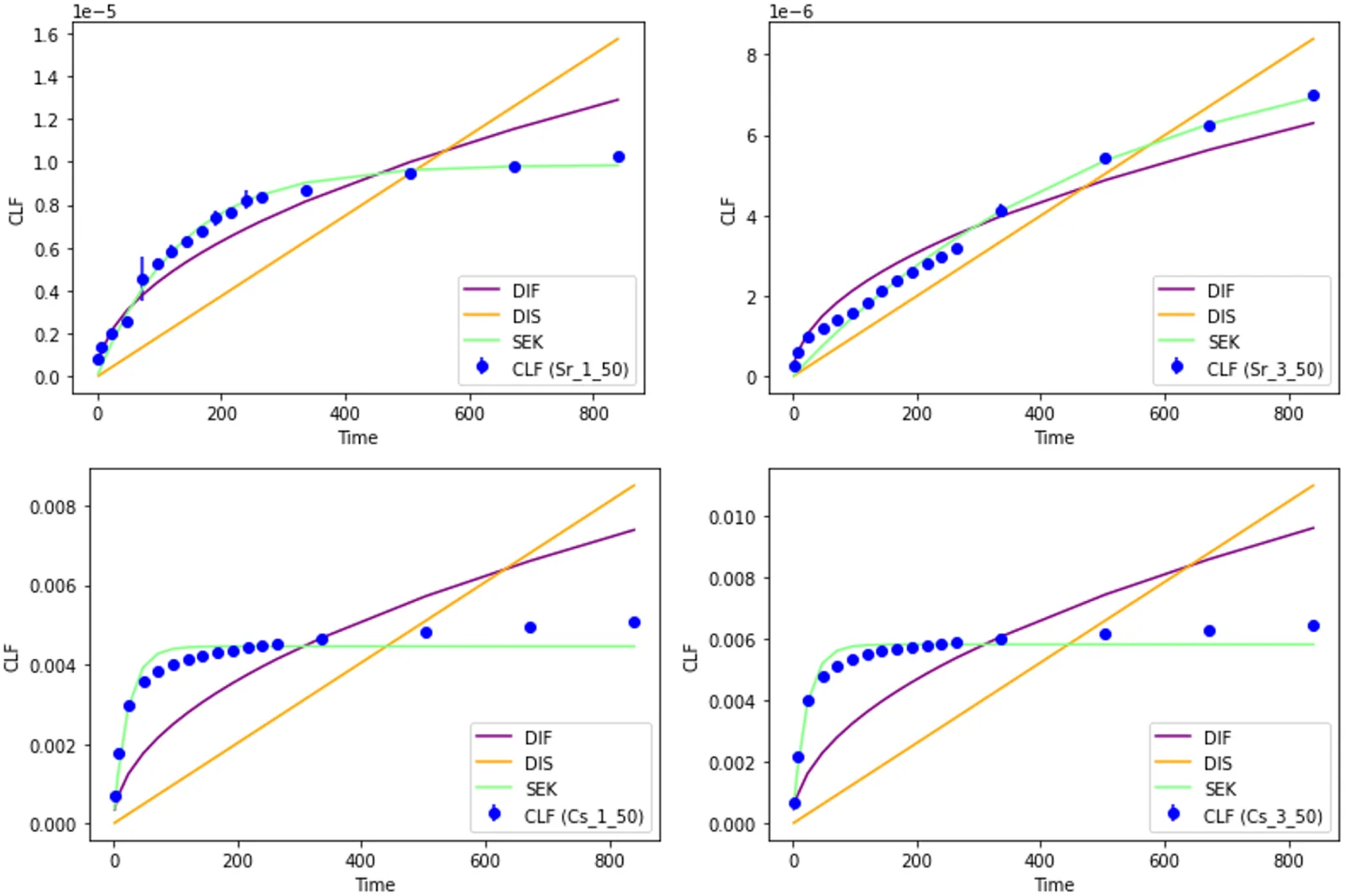
Fits of the single-mechanism mass transport models (diffusion, dissolution and surface exchange kinetics) to the experimental CFL data for Sr (top) and Cs (bottom) during leaching at 50 °C. Symbols denote experimental measurements and solid lines represent model predictions obtained by nonlinear regression.

**Fig. 13:**
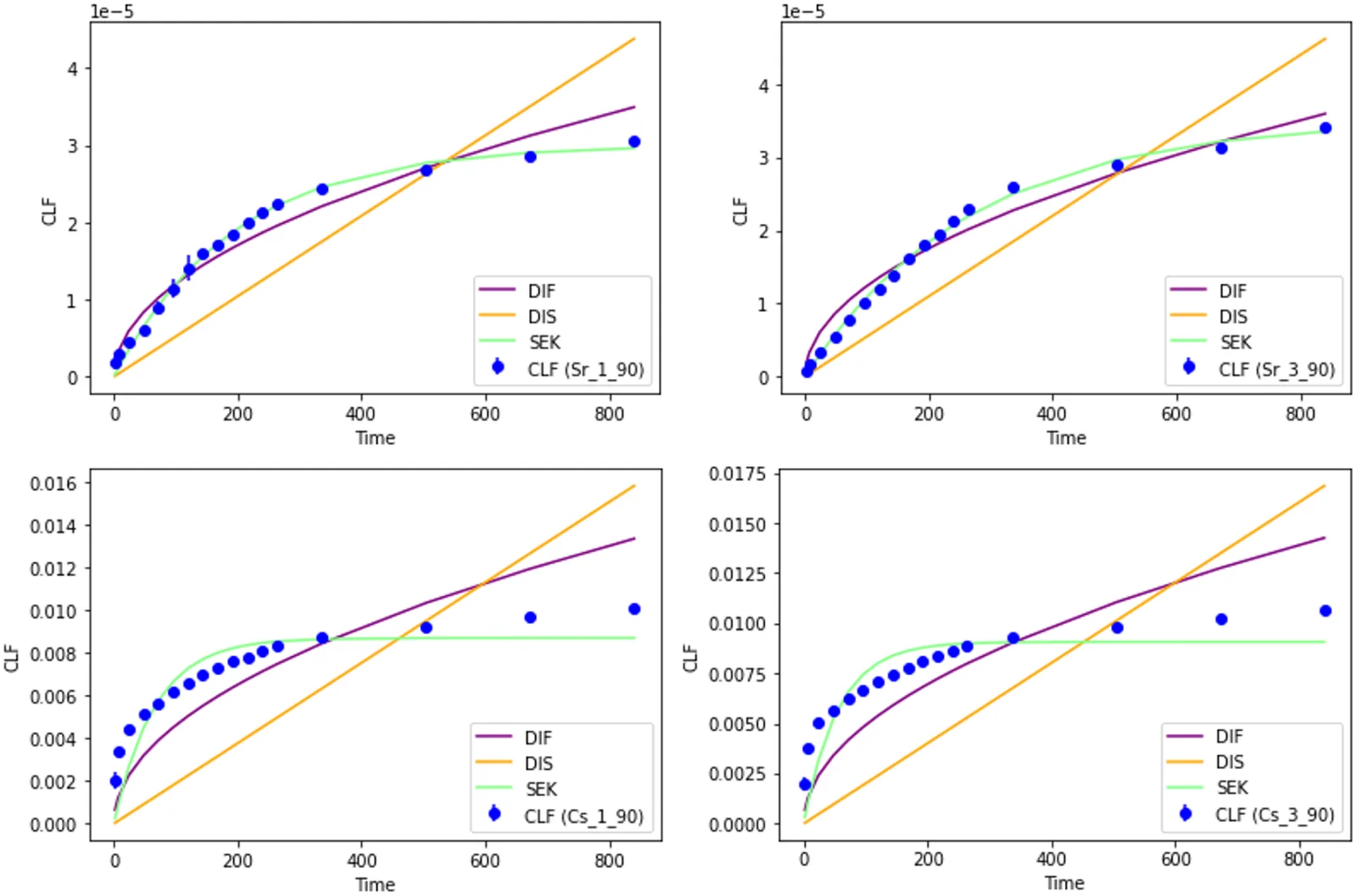
Fits of the single-mechanism mass transport models (diffusion, dissolution and surface exchange kinetics) to the experimental CFL data for Sr (top) and Cs (bottom) during leaching at 90 °C. Symbols denote experimental measurements and solid lines represent model predictions obtained by nonlinear regression.

To determine any changes in the dominating mass transport mechanisms and to assess the goodness of fit for each model, curve fitting was performed using Python's ‘scipy.optimize.curve_fit’ function. Fig. 11–13 illustrate the fits for the data from the 35, 50, and 90 °C leach tests, respectively. The resulting parameters derived from the fit of the models to the CFL and the goodness-of-fit metrics are summarised in the SI.

The diffusion model (DIF) demonstrates a good fit for strontium at all temperatures, with *R*^2^ values exceeding 0.85 and often surpassing 0.9. At 35 °C, the DIF model accurately predicts release behaviour up to approximately 300 hours, after which it underestimates release, failing to capture the plateau. This causes the slightly elevated *χ*_red_^2^ value, suggesting potential under-fitting. A similar trend is observed for the Sr_1_50 sample. However, for the Sr_3_50 sample, the model initially overestimates release then underestimates after 300 hours. Despite this, the overall fit at 50 °C is reasonable, though the high *χ*_red_^2^ values indicate significant under-fitting. At 90 °C, the DIF model has excellent agreement with the experimental release data, achieving *R*^2^ values substantially above 0.9. However, the *χ*_red_^2^ values are relatively high, suggesting that the model does not fully capture the data.

Conversely, the DIF model poorly represents the caesium experimental release at all temperatures. It consistently underestimates the initial release up to 200 hours, and overestimates after, failing to replicate the shape of the release. This suggests the involvement of multiple release mechanisms. Finally, the *χ*_red_^2^ values are high, particularly for the 50 °C samples, showing the inadequacy of the model.

The dissolution model (DIS) consistently provides the poorest fit to the experimental data, failing to reproduce the experimental release. This aligns with the structural analysis, given the absence of any significant structural breakdown due to leaching within the timescale. Dissolution will likely become more relevant towards the wasteforms end of life. The exceptionally high *χ*_red_^2^ values, resulting from large residuals, are indicative of the poor fit.

Finally, the surface exchange kinetics (SEK) model shows the best single-source mechanism fit across most samples, achieving high *R*^2^ values. For strontium, the *R*^2^ values consistently exceed 0.9, reaching over 0.98 at 50 and 90 °C. This suggests that surface exchange significantly contributes to the strontium release. Although *χ*_red_^2^ values are generally low, they are still suboptimal, indicating potential over- or under-fitting.

Overall, some clear trends emerge across the temperature range. Firstly, surface exchange kinetics always contributes to both caesium and strontium release, indicating significant cation–leachate interactions, especially in the first 200 hours. Secondly, dissolution does not contribute significantly to release, consistent with the solid state characterisation and geopolymer durability. Finally, diffusion becomes more dominant at higher temperatures. This is expected due to increased particle kinetic energy. However, no single mechanism fully explained the experimental release, suggesting a combined mass transport mechanism.

#### 4.6.2 Combined source mass transport models

**Fig. 14:**
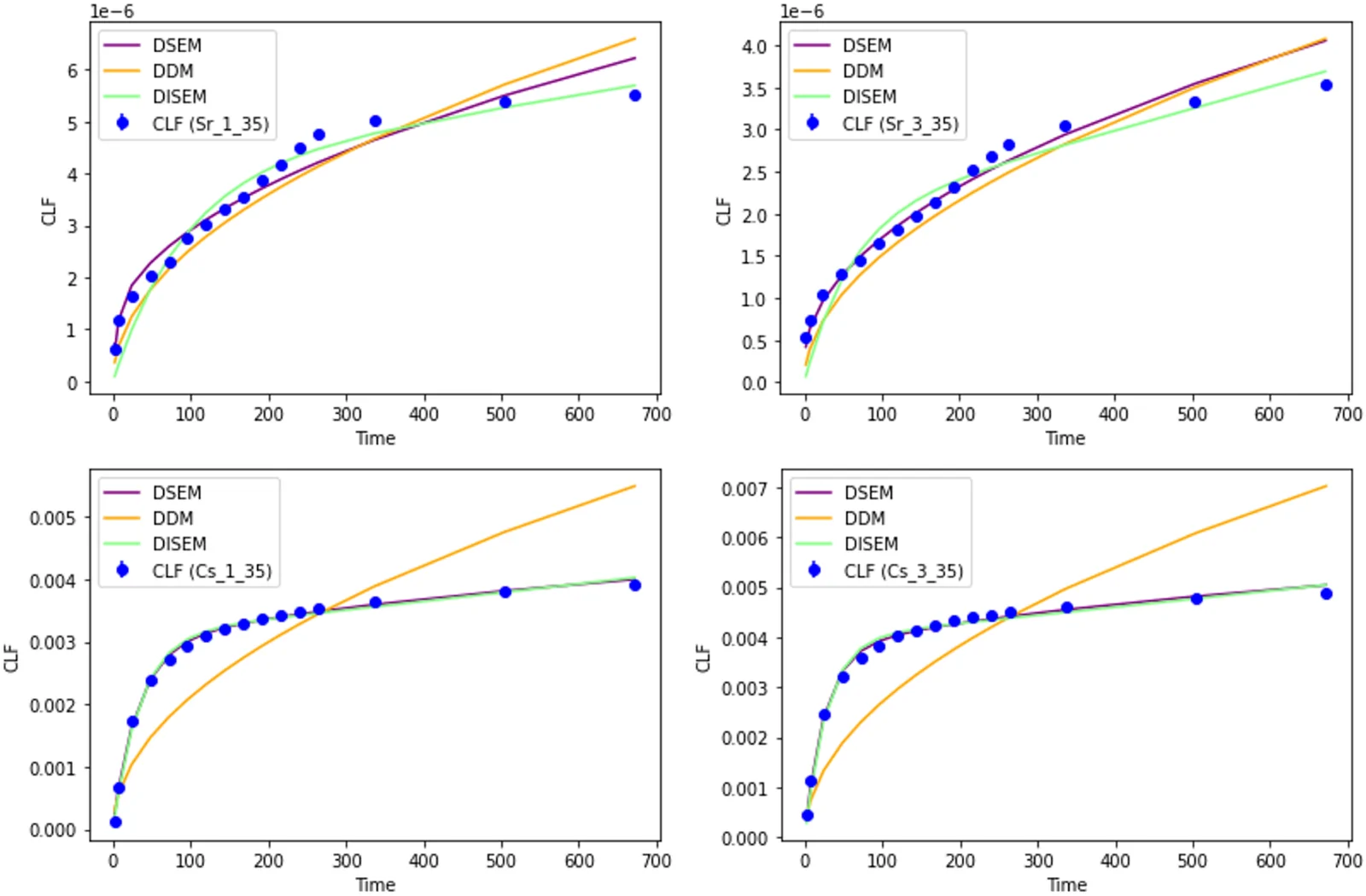
Fits of the combined-mechanism mass transport models to the experimental CFL data for Sr (top) and Cs (bottom) during leaching at 35 °C. Symbols denote experimental measurements and solid lines represent model predictions obtained by nonlinear regression.

**Fig. 15:**
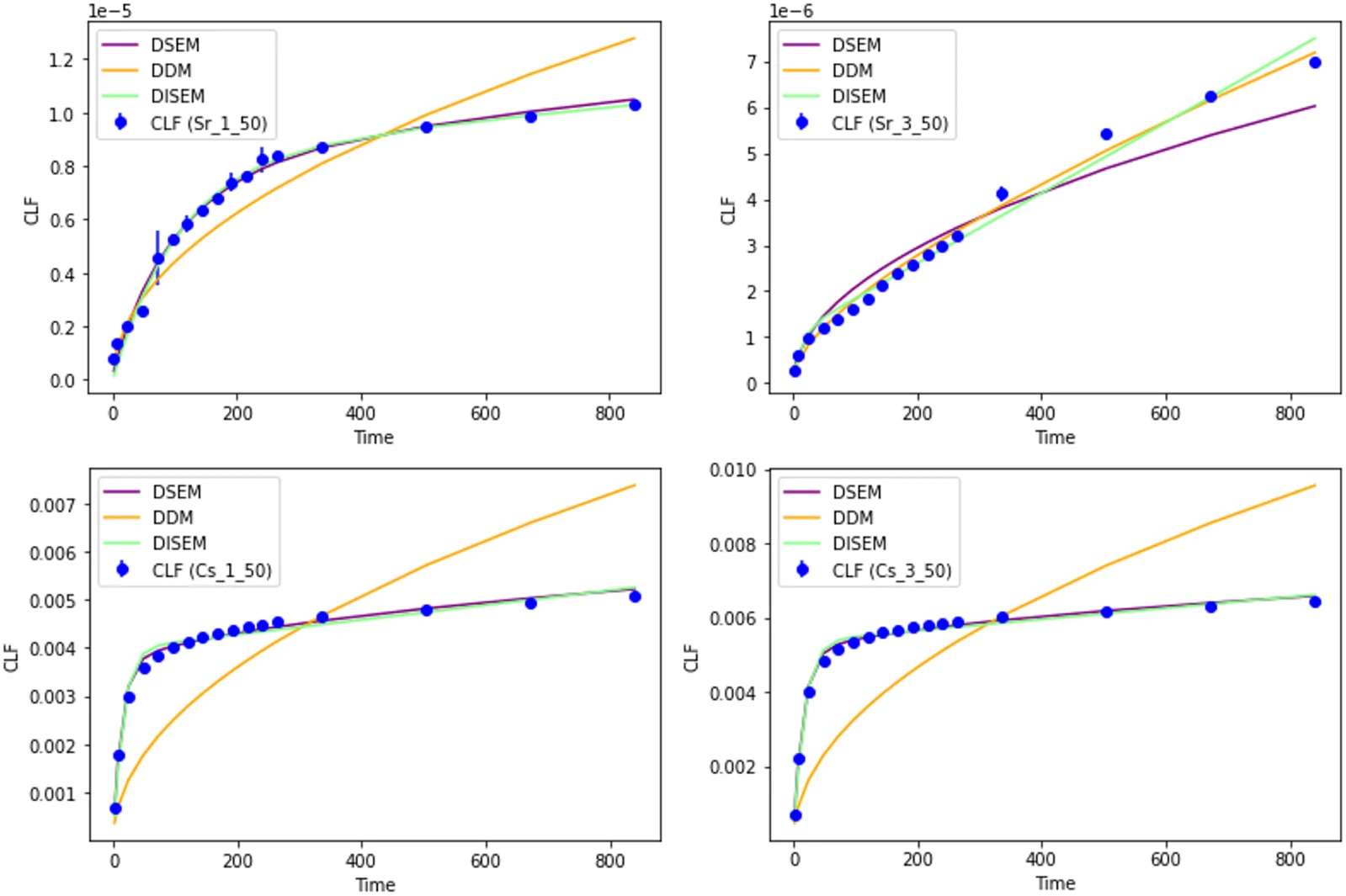
Fits of the combined-mechanism mass transport models to the experimental CFL data for Sr (top) and Cs (bottom) during leaching at 50 °C. Symbols denote experimental measurements and solid lines represent model predictions obtained by nonlinear regression.

**Fig. 16:**
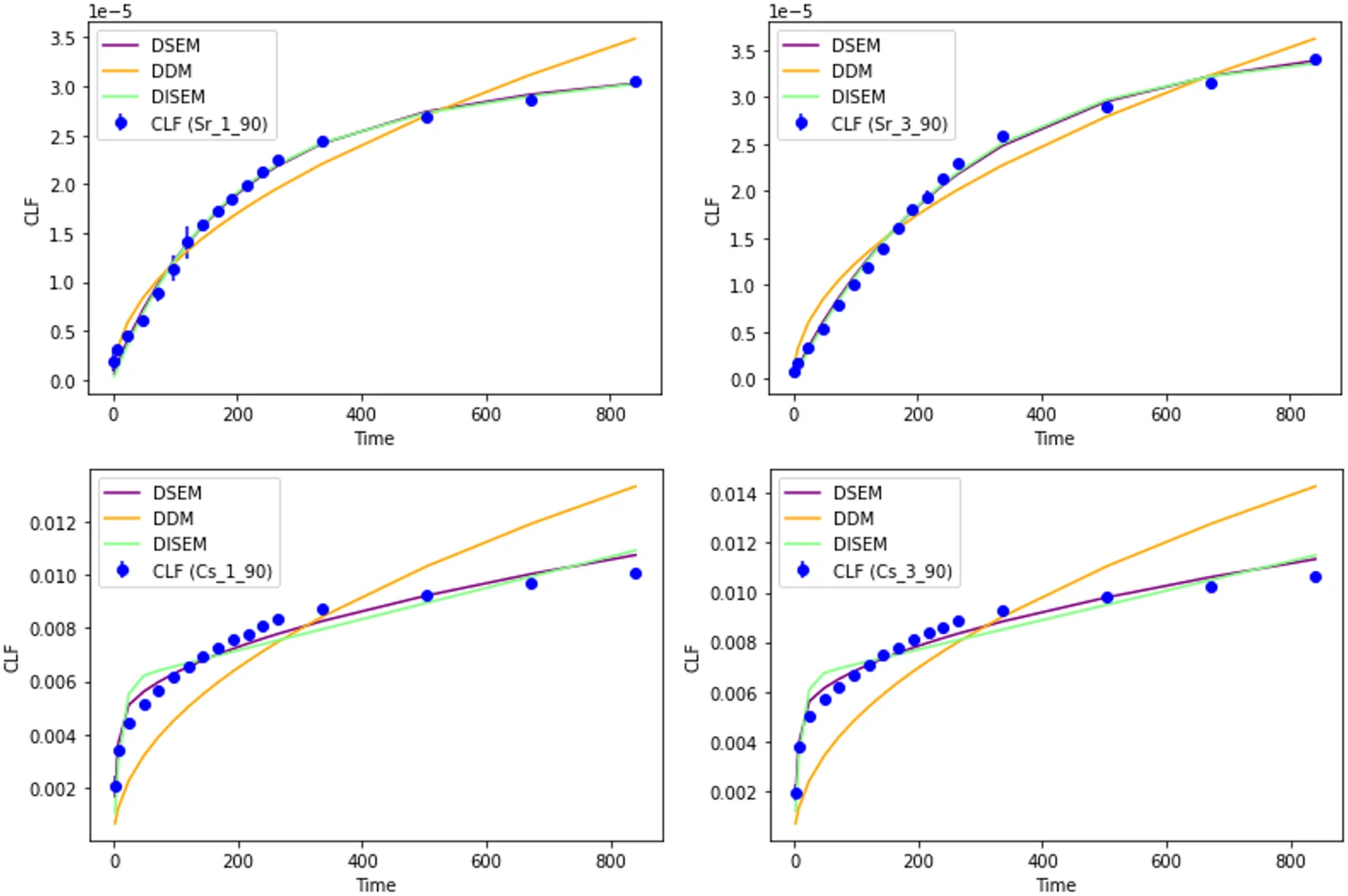
Fits of the combined-mechanism mass transport models to the experimental CFL data for Sr (top) and Cs (bottom) during leaching at 90 °C. Symbols denote experimental measurements and solid lines represent model predictions obtained by nonlinear regression.

To confirm the contribution of the different mass transport mechanisms to the release of caesium and strontium from the geopolymer, individual fits were superimposed (see Section 3) and subsequently fit to the experimental CFL release data. The fitting was completed using Python's ‘scipy.optimize.differential_evolution’ function, a stochastic function which finds global maxima in large areas of candidate space. The resulting model parameters and the goodness of fit metrics are shown in the SI and the corresponding plots are displayed in Fig. 14–16.

Visual inspection of the fitted curves reveal that the combined models provide a significantly more accurate representation of the experimental release data across all temperatures. The diffusion–surface exchange model (DSEM) demonstrates an excellent fit for strontium release data at all temperatures, achieving *R*^2^ values exceeding 0.94. However, at 35 °C, the DSEM model does begin to overestimate experimental release for *t* > 400 hours, and for Sr_3_50, it begins to underestimate release. The *χ*_red_^2^ value, while indicative of a good fit at 35 °C and 90 °C, is suboptimal at 50 °C. For caesium, the DSEM model also exhibits good agreement with the experimental data, with *R*^2^ values above 0.9. However, at 50 °C and 90 °C, the *χ*_red_^2^ values are unacceptably high, suggesting an inadequate model.

Conversely, the diffusion-dissolution model (DDM) consistently provides the poorest fit among the combined model for almost all samples, excluding Sr_1_50. The DDM model fails to accurately predict the characteristic curve of the experimental release data, resulting in substantially high *χ*_red_^2^ values, indicating poor agreement with experimental observations.

The dissolution–surface exchange model (DISEM) also effectively captures the experimental release, with predictions almost identical to those of the DSEM model. For strontium-containing samples, the *χ*_red_^2^ values generally fall within an acceptable range, with the exception of the Sr_3_50 sample. However, for caesium they are generally slightly higher than acceptable.

Overall, the findings confirm that mass transport in this system is governed by multi-parametric diffusion and surface exchange kinetics. Dissolution processes are unlikely to play a significant role over the experimental timescale, given the intrinsic durability of geopolymers.^3^ As predicted by the Arrhenius principle, diffusion mechanisms become increasingly dominant with rising temperature,^32^ leading to the higher release rates at 90 °C compared with the lower temperatures.

#### 4.6.3 Statistical analysis

To further understand the goodness of fit, root cause of the high *χ*_red_^2^, which can indicate the model is not fully capturing the data, values was assessed. This can be caused by three potential issues: incorrect measurement of uncertainties, a poor model fit, or outliers in the data.^17^ Firstly, all data was taken in triplicate to allow the determination of outliers, which could be removed from the dataset. Secondly, the SD was scaled by a factor of 10 to assess if the small errors were a root cause, which did reduce the *χ*_red_^2^ values but they still exceeded 1. Finally, the residuals were assessed to determine how well the model predicted the experimental release. The residuals should be random around the horizontal line if the model represents the data well, as calculated by eqn (15). A negative residual indicates that the model over-predicted the experimental data and a positive residual indicates that the model under-predicted the experimental data. The residuals plots for all data is in SI.

15 Residuals = observed value − predicted value

In the single-source models, the behaviour of strontium (Sr) residuals varied across different temperatures. At 35 °C, residuals were initially positive but became negative towards the end. Among the models, SEK exhibited the smallest deviation and remained relatively balanced around the horizontal line, whereas DIS performed the worst. At 50 °C, the 1% Sr residuals were similar to those observed at 35 °C. However, in the residuals of 3% Sr, SEK showed slight improvement, while DIF performed worse. At 90 °C, DIS exhibited a similar pattern to its performance at lower temperatures, while SEK performed well. DIF showed some improvement but still oscillated between negative and positive values. However, for Cs, all models displayed a similar trend. Both DIS and DIF initially had positive residuals before turning negative, with DIS performing the worst. SEK maintained a more even distribution around the horizontal line but still exhibited some degree of residual trend.

In the combined-source models, the behaviour of the strontium fits at 35 °C followed similar trends across all models at both the 1% and 3% levels. At 50 °C and 90 °C, DSEM and DISEM displayed more random residuals, whereas DDM continued to exhibit a clear trend. For caesium, DISEM and DSEM had small and relatively evenly distributed residuals, indicating better performance. In contrast, DIS showed a clear residual trend and performed poorly. At 90 °C, the performance of the models declined further, with more noticeable trends in the residuals.

The reduced *χ*_red_^2^ statistic is sensitive to the assumed experimental uncertainty and the suitability of the error model. In this work, experimental uncertainties were small because each data point represents the mean of triplicate measurements performed under controlled ASTM C1308 conditions. Consequently, even relatively small deviations between the analytical models and experimental data produce elevated *χ*_red_^2^ values. Furthermore, the fitting was performed using an unweighted least-squares approach, which assumes constant variance across all time points despite the CFL spanning several orders of magnitude. Finally, the analytical expressions are simplified representations of a complex process involving diffusion, surface exchange, and local structural effects, and therefore cannot be expected to capture all experimental variability. For these reasons, model performance was assessed using a combination of *R*^2^, residual analysis, and physical consistency rather than *χ*_red_^2^ alone. While this is a limitation and future studies could benefit from collecting additional data points to improve model performance, the models are not intended to represent long-term behaviour beyond the experimental timescale, but rather as phenomenological descriptions of the experimental release behaviour within the 35 days period.

#### 4.6.4 Temperature dependency

**Fig. 17:**
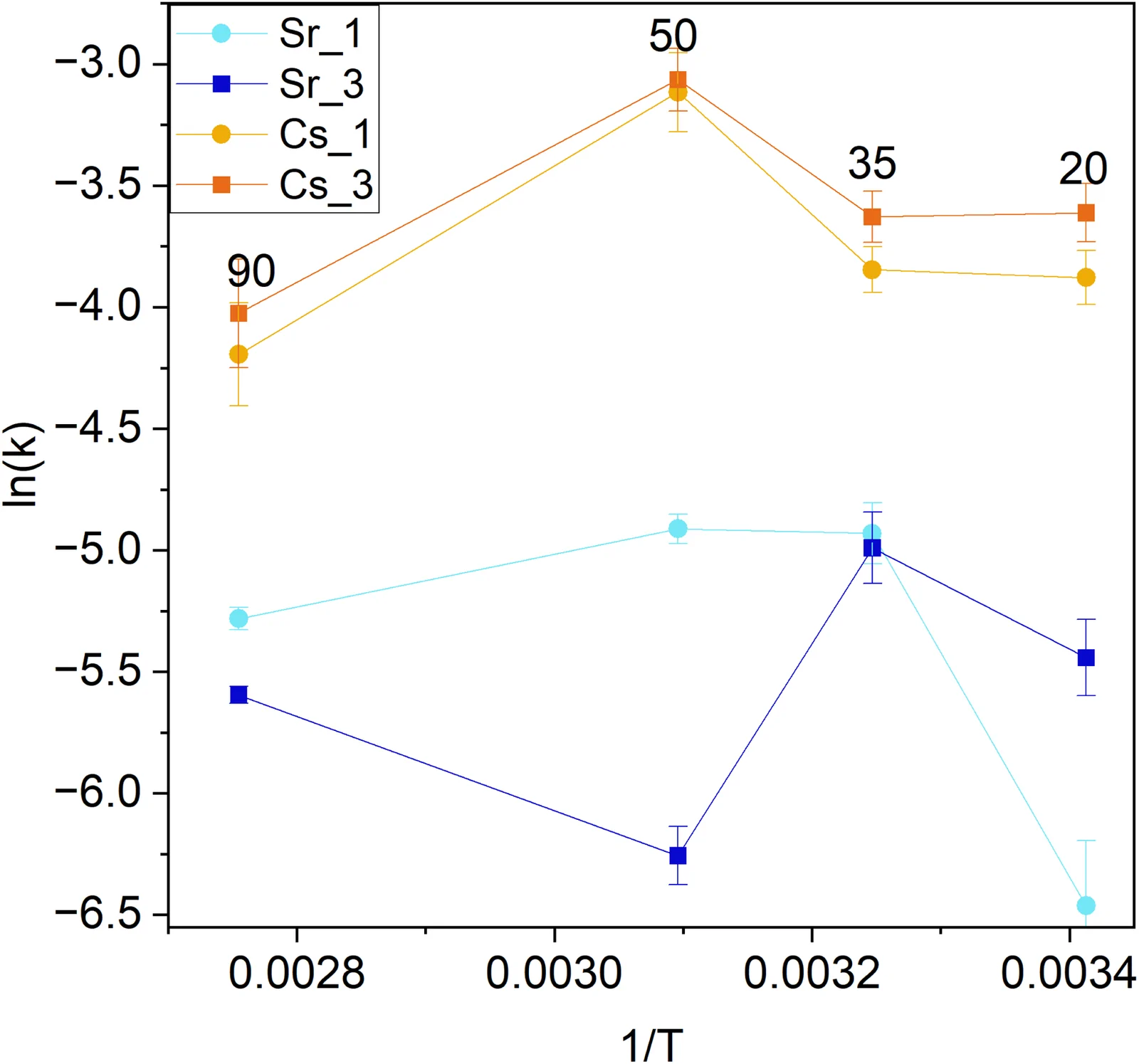
Temperature-dependent behaviour of the rate constant. ln(*k*) values calculated from the models.

To determine whether the experimental release exhibits a linear temperature dependency, a plot of 1/*T vs.* ln(*k*) (Fig. 17) was constructed to assess Arrhenius behaviour (eqn (16)). Given that surface exchange kinetic mechanisms were identified as the dominant process, the rate constant was extracted from the SEK model. Visual inspection of the plot indicates that the data does not follow a linear trend and deviates from Arrhenius behaviour.16
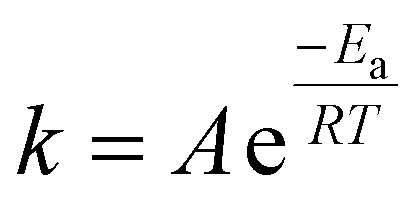
where *k* is the rate constant (h^−1^), *A* is the pre-exponential factor (Arrhenius constant, h^−1^), *E*_a_ is the activation energy (J mol^−1^ K^−1^), *R* is the universal gas constant (J mol^−1^), and *T* is the absolute temperature (K).

For the caesium-containing samples, a consistent trend can be observed where the values at 20 °C and 35 °C are of similar magnitude. This indicates temperature is not having a significant effect on the surface exchange kinetics. At 50 °C, ln(*k*) increases, suggesting that temperature begins to have an impact. Unexpectedly, however, ln(*k*) decreases at 90 °C. This trend aligns with findings from the mass transport mechanism analysis, where SEK dominates at lower temperatures, before diffusion effects become more significant at higher temperatures, diminishing the impact of temperature on the kinetics.

In the 1% strontium sample, a similar trend is observed, although the temperature dependency is more pronounced between 20 °C and 35 °C, and diffusion effects are less significant. This may explain the visual reduction in the magnitude of the inversion on the CFL plot at 35 °C compared with 20 °C. This suggests that a certain mechanism may not initiate until temperatures exceed 20 °C. However, the 3% strontium sample does not exhibit a clear trend. Although an increase is observed between 20 °C and 35 °C, ln(*k*) drops significantly at 50 °C before rising again at 90 °C. The cause of this is unclear and may be attributed to an alternative mass transport mechanism or experimental outliers.

Overall, the observed temperature dependency does not conform to the Arrhenius principle, indicating the mass transport is not governed by a single activation energy across the temperature range. This is expected, as multiple mechanisms are contributing the complex mass transport behaviour. Other principles, such as the Vogel–Tammann–Fulcher (VTF) equation, Williams–Landel–Ferry (WLF) equation, or a general, empirical approach may define the behaviour more explicitly.

## 5 Conclusions

This study evaluated the impact of elevated temperatures (35, 50, and 90 °C) on the performance and mass transport mechanisms of Cs- and Sr-loaded geopolymer wasteforms during leaching, furthering the safety case for their use in geological disposal facilities. The geopolymers investigated exhibited excellent thermal stability and maintained superior radionuclide retention performance compared to conventional cementitious systems under simulated repository temperature extremes. Therefore, under the parameters studied in this work, geopolymers are an excellent candidate wasteform material for the disposal of Cs-137 and Sr-90.

Across all tested temperatures, all geopolymer samples achieved acceptable leachability indices (Li > 6), confirming their high resistance to radionuclide release. A clear and pronounced temperature dependency was observed, resulting in a nearly threefold increase in Cs release and an order of magnitude increase in Sr release at 90 °C compared to lower temperatures. This increase is partially attributed to the enhanced solubility of secondary phases like SrCO_3_ at higher temperatures.

Structural analysis utilising XRD, FTIR, and NMR confirmed that the amorphous K–A–S–H gel structure remained consistently stable across all tested temperatures (35, 50, and 90 °C), indicating that predicted repository thermal fluctuations will not compromise the bulk stability of the geopolymer matrix. Element-specific X-ray absorption near-edge structure (XANES) analysis indicated that Sr undergoes some structural reordering upon leaching at elevated temperatures, transitioning towards environments similar to SrCO_3_ or Sr(OH)_2_.

Mass transport modelling confirmed that Cs and Sr release is governed by a complex, multi-parametric mechanism dominated by diffusion and surface exchange kinetics. As predicted by kinetic principles, diffusion mechanisms became increasingly dominant with rising temperature, directly contributing to the higher release rates observed at 90 °C. The mass transport rate constants did not conform to the simple Arrhenius principle, suggesting the process is governed by multiple overlapping mechanisms rather than a single activation energy across the investigated temperature range (20–90 °C).

Future work should explore alternative kinetic models, such as the Vogel–Tammann–Fulcher equation or the Williams–Landel–Ferry equation, to more explicitly define the complex, non-Arrhenius temperature dependency of the mass transport behaviour.

## Conflicts of interest

There are no conflicts to declare.

## Supplementary Material

TA-014-D6TA01864E-s001

## Data Availability

All data supporting this article have been included as part of the main manuscript. Supplementary information (SI) is available. See DOI: https://doi.org/10.1039/d6ta01864e.
