# Supplementary material for: Effect of temperature on Sr and Cs incorporation and mass transport mechanisms in geopolymer cement wasteforms
Source: J Mater Chem A Mater. 2026 Sep 2;14(59):41295–319. doi: 10.1039/d6ta01864e (PMC13572939; doi:10.1039/d6ta01864e)
Supplement: Supplementary file 1 [file TA-014-D6TA01864E-s001.pdf]

Supplementary Information (SI) for Journal of Materials Chemistry A.
This journal is © The Royal Society of Chemistry 2026

1 Supplementary information

1.1 Si and Al CFL

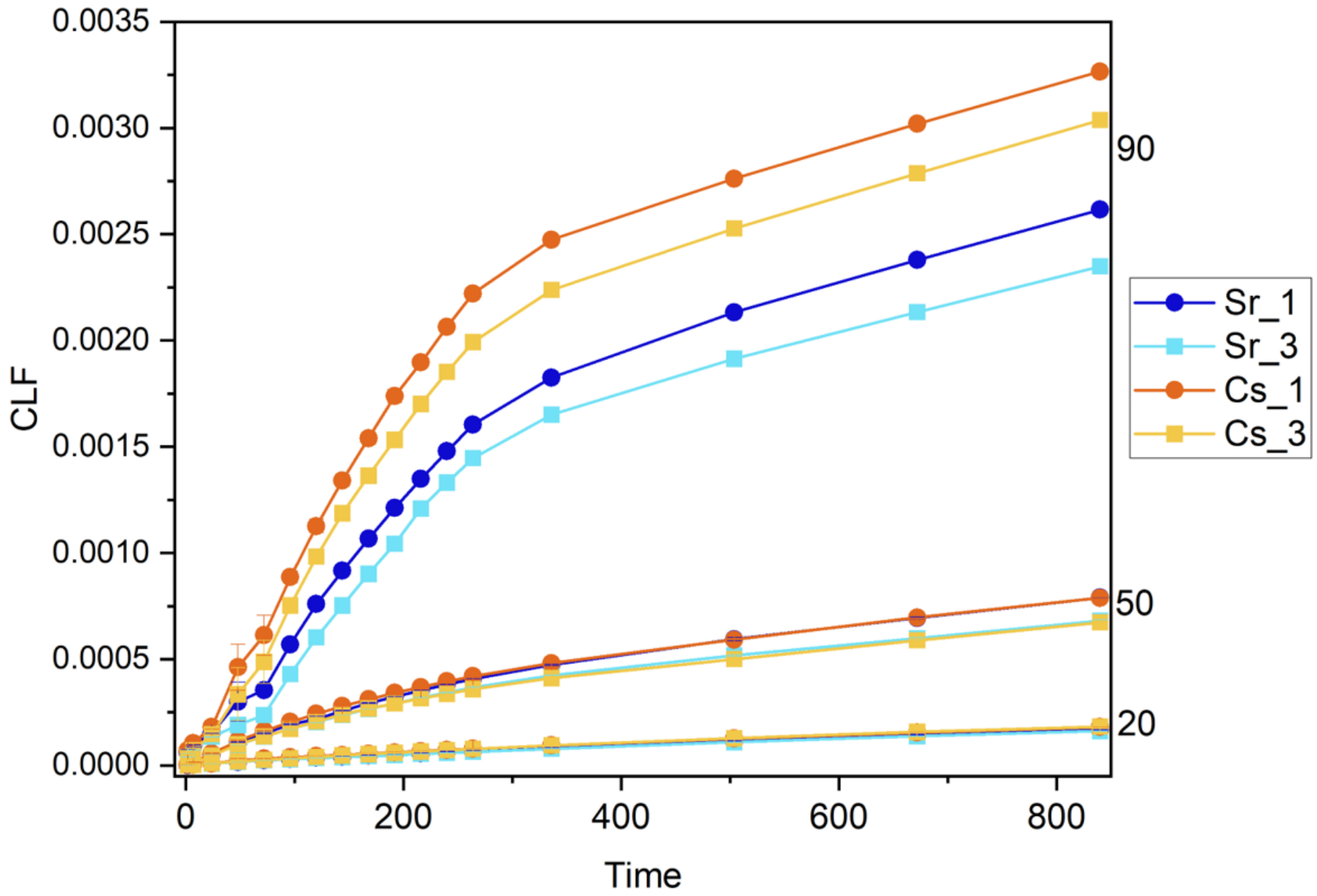

Figure 1: CFL for Al across all 20, 50 and 90 °C. 35°C is omitted due to an incomplete dataset.

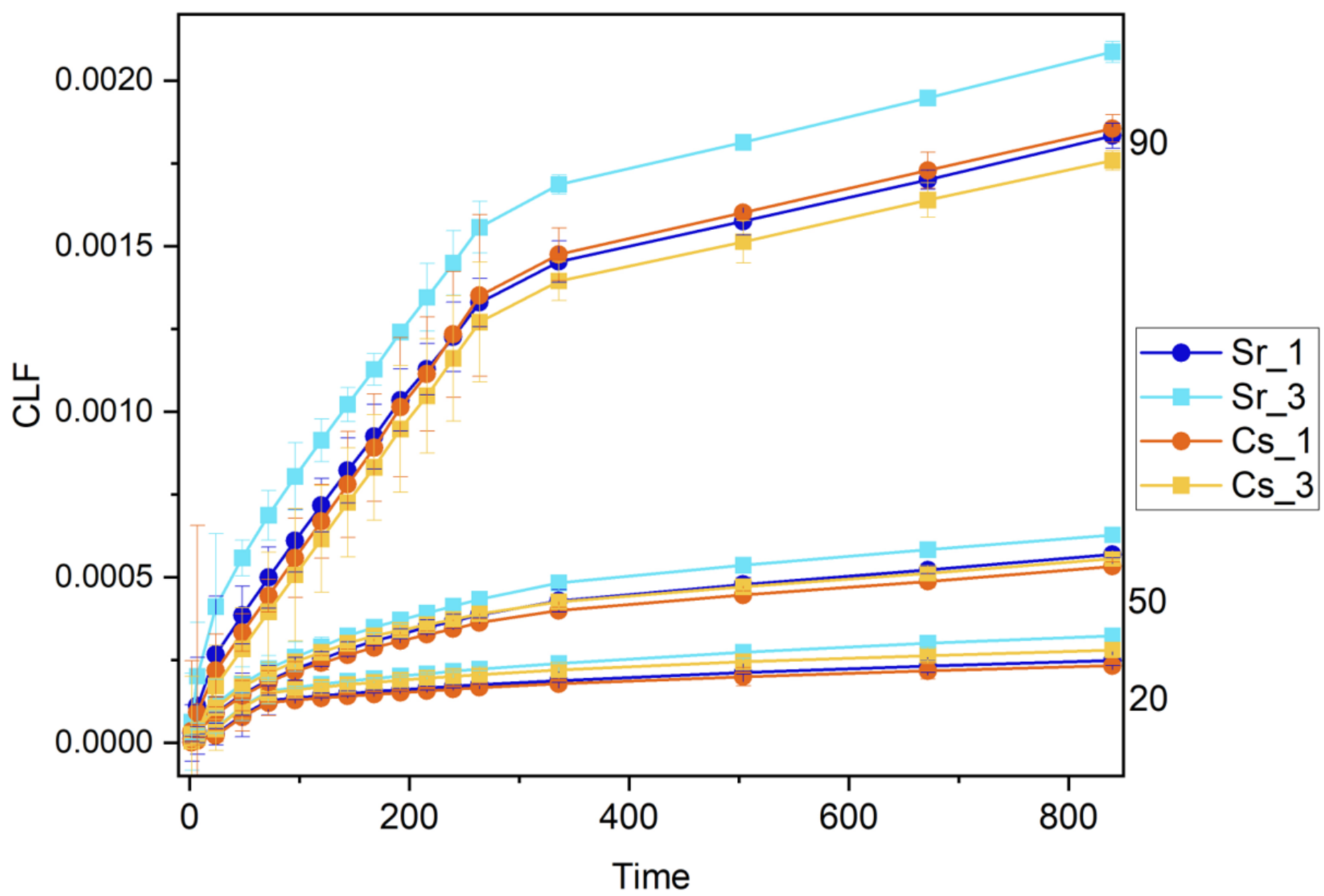

Figure 2: CFL for Si across all 20, 50 and 90 °C. 35°C is omitted due to an incomplete dataset.

1.2 Single source mass transport model fitting data

Table 1: Associated variables for each single source mass transport model as calculated from 'scipy.optimize.curve_fit' on Python programming software.

**Sample**	**Model**	**Variable**	$\mathbf{R^2}$	$\chi^2_{\mathbf{red}}$
Sr_1_35	DIF	$D = 1.74 \times 10^{-16} \pm 1.1 \times 10^{-17}$	0.921	4.17
	DIS	$U = 7.27 \times 10^{-10} \pm 8.3 \times 10^{-11}$	0.357	46.1
	SEK	$C = 5.45 \times 10^{-6} \pm 2.8 \times 10^{-7}$<br>$k = 0.00722 \pm 0.00091$	0.947	1.38
Sr_3_35	DIF	$D = 6.54 \times 10^{-17} \pm 3.6 \times 10^{-18}$	0.937	5.20
	DIS	$U = 4.47 \times 10^{-10} \pm 4.9 \times 10^{-11}$	0.389	61.1
	SEK	$C = 3.39 \times 10^{-6} \pm 2.1 \times 10^{-7}$<br>$k = 0.00681 \pm 0.0010$	0.923	1.93
Cs_1_35	DIF	$D = 1.15 \times 10^{-10} \pm 1.4 \times 10^{-11}$	0.606	166.2
	DIS	$U = 5.66 \times 10^{-7} \pm 8.7 \times 10^{-8}$	-0.372	977
	SEK	$C = 0.00355 \pm 7.0 \times 10^{-5}$<br>$k = 0.0214 \pm 0.0020$	0.972	10.8
Cs_3_35	DIF	$D = 1.88 \times 10^{-10} \pm 2.5 \times 10^{-11}$	0.462	713
	DIS	$U = 7.18 \times 10^{-7} \pm 1.2 \times 10^{-7}$	-0.708	4460
	SEK	$C = 0.00446 \pm 8.7 \times 10^{-5}$<br>$k = 0.0266 \pm 0.0028$	0.964	39.4
Sr_1_50	DIF	$D = 5.08 \times 10^{-16} \pm 4.1 \times 10^{-17}$	0.869	7.01
	DIS	$U = 1.07 \times 10^{-9} \pm 1.4 \times 10^{-10}$	0.280	43.1
	SEK	$C = 9.86 \times 10^{-6} \pm 2.2 \times 10^{-7}$<br>$k = 0.00736 \pm 0.00044$	0.986	0.173
Sr_3_50	DIF	$D = 1.21 \times 10^{-16} \pm 7.8 \times 10^{-18}$	0.954	12.2
	DIS	$U = 5.71 \times 10^{-10} \pm 3.0 \times 10^{-11}$	0.918	37.7
	SEK	$C = 8.66 \times 10^{-6} \pm 6.3 \times 10^{-7}$<br>$k = 0.00192 \pm 0.00023$	0.985	0.786
Cs_1_50	DIF	$D = 1.67 \times 10^{-10} \pm 2.7 \times 10^{-11}$	-0.523	26.0
	DIS	$U = 5.80 \times 10^{-7} \pm 1.0 \times 10^{-7}$	-1.59	247

	SEK	C = 0.00446 ± 0.00010<br>k = 0.0.0444 ± 0.0072	0.912	887
Cs_3_50	DIF	$D = 2.82 \times 10^{-10} \pm 4.9 \times 10^{-11}$	-0.140	147
	DIS	$U = 7.49 \times 10^{-7} \pm 1.4 \times 10^{-7}$	-1.67	432
	SEK	C = 0.0.00583 ± 0.00010<br>k = 0.0.0457 ± 0.0.00591	0.950	1020
Sr_1_90	DIF	$D = 3.74 \times 10^{-15} \pm 2.1 \times 10^{-16}$	0.948	10.6
	DIS	$U = 2.98 \times 10^{-9} \pm 3.1 \times 10^{-10}$	0.565	103
	SEK	$C = 3.00 \times 10^{-5} \pm 6.1 \times 10^{-7}$<br>k = 0.00508 ± 0.0.00023	0.993	1.13
Sr_3_90	DIF	$D = 3.95 \times 10^{-15} \pm 2.1 \times 10^{-16}$	0.964	12.5
	DIS	$U = 3.14 \times 10^{-9} \pm 2.7 \times 10^{-10}$	0.736	217
	SEK	$C = 3.51 \times 10^{-5} \pm 6.12 \times 10^{-7}$<br>k = 0.0.00372 ± 0.000131	0.997	2.43
Cs_1_90	DIF	$D = 5.45 \times 10^{-10} \pm 6.56 \times 10^{-11}$	0.508	2.50
	DIS	$U = 1.08 \times 10^{-6} \pm 1.6 \times 10^{-7}$	-0.700	7.67
	SEK	C = 0.0.000870 ± 0.000455<br>k = 0.0151 ± 0.0032	0.772	0.485
Cs_3_90	DIF	$D = 6.23 \times 10^{-10} \pm 7.8 \times 10^{-11}$	0.448	1.87
	DIS	$U = 1.15 \times 10^{-6} \pm 1.8 \times 10^{-7}$	-0.813	9.69
	SEK	C = 0.00908 ± 0.00045<br>k = 0.0179 ± 0.0040	0.760	0.641

1.3 Combined source mass transport model fitting data

Table 2: Model fit details for 35 °C as calculated from 'scipy.optimize.differential_evolution' package.

Sample	Model	Parameter	R^2	χ^2
Sr_1_35	DSEM	$D = 1.11 \times 10^{-16} \pm 3.5 \times 10^{-17}$ $C = 8.36 \times 10^{-7} \pm 5.0 \times 10^{-7}$ $K = 0.195 \pm 0.37$	0.957	3.94
	DDM	$D = 1.67 \times 10^{-16} \pm 0.0$ $U = 1.79 \times 10^{-14} \pm 2.7 \times 10^{-11}$	0.908	6.08
	DISEM	$U = 1.40 \times 10^{-10} \pm 6.7 \times 10^{-11}$ $C = 4.05 \times 10^{-6} \pm 5.2 \times 10^{-7}$ $K = 0.0109 \pm 0.29$	0.944	1.85
Sr_3_35	DSEM	$D = 5.55 \times 10^{-17} \pm 7.8 \times 10^{-17}$ $C = 2.46 \times 10^{-7} \pm 4.30 \times 10^{-7}$ $K = 0.996 \pm 0.228$	0.963	4.01
	DDM	$D = 5.55 \times 10^{-17} \pm 2.1 \times 10^{-9}$ $U = 2.29 \times 10^{-11} \pm 2.8 \times 10^{-10}$	0.901	6.09
	DISEM	$U = 2.04 \times 10^{-16} \pm 4.63 \times 10^{-11}$ $C = 2.72 \times 10^{-6} \pm 3.56 \times 10^{-7}$ $K = 0.0165 \pm 0.271$	0.930	2.06
Cs_1_35	DSEM	$D = 7.30 \times 10^{-12} \pm 2.38 \times 10^{-12}$ $C = 0.00262 \pm 0.000118$ $K = 0.0310 \pm 0.00301$	0.997	1.22
	DDM	$D = 1.15 \times 10^{-10} \pm 1.75 \times 10^{-11}$ $U = 1.69 \times 10^{-10} \pm 1.63 \times 10^{-10}$	0.560	178
	DISEM	$U = 7.98 \times 10^{-8} \pm 4.06 \times 10^{-8}$ $C = 0.00309 \pm 0.000339$ $K = 0.0295 \pm 0.302$	0.995	2.35
Cs_3_35	DSEM	$D = 1.08 \times 10^{-11} \pm 5.06 \times 10^{-12}$ $C = 0.00337 \pm 0.000194$ $K = 0.0398 \pm 0.00512$	0.995	9.30
	DDM	$D = 1.89 \times 10^{-10} \pm 3.20 \times 10^{-11}$ $U = 3.54 \times 10^{-10} \pm 1.70 \times 10^{-10}$	0.381	773
	DISEM	$U = 9.33 \times 10^{-8} \pm 4.56 \times 10^{-8}$ $C = 0.00395 \pm 0.000345$ $K = 0.0361 \pm 0.260$	0.989	15.8

Table 3: Model fit details for 50 °C as calculated from 'scipy.optimize.differential_evolution' package.

**Sample**	**Model**	**Parameter**	$\mathbf{R^2}$	χ^2
Sr_1_50	DSEM	$D = 5.55 \times 10^{-17} \pm 9.24 \times 10^{-17}$<br>$C = 6.23 \times 10^{-6} \pm 9.91 \times 10^{-7}$<br>$K = 0.00959 \pm 0.0237$	0.990	0.252
	DDM	$D = 5.00 \times 10^{-16} \pm 6.11 \times 10^{-17}$<br>$U = 2.44 \times 10^{-15} \pm 3.13 \times 10^{-11}$	0.859	7.39
	DISEM	$U = 1.33 \times 10^{-10} \pm 7.75 \times 10^{-11}$<br>$C = 8.34 \times 10^{-6} \pm 8.27 \times 10^{-7}$<br>$K = 0.00931 \pm 0.340$	0.989	0.149
Sr_3_50	DSEM	$D = 1.11 \times 10^{-16} \pm 1.09 \times 10^{-17}$<br>$C = 5.15 \times 10^{-11} \pm 1.12 \times 10^{-7}$<br>$K = 0.0955 \pm 0.00978$	0.944	2034
	DDM	$D = 5.55 \times 10^{-17} \pm 1.10 \times 10^{-17}$<br>$U = 1.99 \times 10^{-10} \pm 4.64 \times 10^{-11}$	0.992	121
	DISEM	$U = 4.39 \times 10^{-10} \pm 5.88 \times 10^{-11}$<br>$C = 1.07 \times 10^{-6} \pm 5.63 \times 10^{-7}$<br>$K = 0.0738 \pm 0.318$	0.984	531
Cs_1_50	DSEM	$D = 9.80 \times 10^{-12} \pm 1.95 \times 10^{-12}$<br>$C = 0.00343 \pm 0.0000984$<br>$K = 0.0761 \pm 0.0123$	0.991	61.7
	DDM	$D = 1.66 \times 10^{-10} \pm 3.84 \times 10^{-11}$<br>$U = 6.89 \times 10^{-11} \pm 1.51 \times 10^{-10}$	-0.285	2667
	DISEM	$U = 8.75 \times 10^{-8} \pm 3.04 \times 10^{-8}$<br>$C = 0.00397 \pm 0.000364$<br>$K = 0.0652 \pm 0.267$	0.979	136
Cs_3_50	DSEM	$D = 9.79 \times 10^{-12} \pm 2.37 \times 10^{-12}$<br>$C = 0.00480 \pm 0.000122$<br>$K = 0.0669 \pm 0.00919$	0.994	60.4
	DDM	$D = 2.80 \times 10^{-10} \pm 5.87 \times 10^{-11}$<br>$U = 1.42 \times 10^{-10} \pm 2.99 \times 10^{-10}$	-0.379	15362
	DISEM	$U = 8.63 \times 10^{-8} \pm 3.90 \times 10^{-8}$<br>$C = 0.00535 \pm 0.000378$<br>$K = 0.0599 \pm 0.234$	0.987	108

Table 4: Model fit details for 90 °C as calculated from 'scipy.optimize.differential_evolution' package.

Sample	Model	Parameter	$\mathbf{R^2}$	χ^2
Sr_1_90	DSEM	$D = 1.67 \times 10^{-16} \pm 1.20 \times 10^{-15}$<br>$C = 2.32 \times 10^{-5} \pm 8.57 \times 10^{-6}$<br>$K = 0.00547 \pm 0.159$	0.994	1.28
	DDM	$D = 3.72 \times 10^{-15} \pm 3.62 \times 10^{-16}$<br>$U = 1.47 \times 10^{-13} \pm 2.65 \times 10^{-10}$	0.948	25.0
	DISEM	$U = 3.25 \times 10^{-10} \pm 3.15 \times 10^{-10}$<br>$C = 2.56 \times 10^{-5} \pm 2.63 \times 10^{-6}$<br>$K = 0.00608 \pm 0.308$	0.993	1.43
Sr_3_90	DSEM	$D = 5.55 \times 10^{-17} \pm 1.94 \times 10^{-15}$<br>$C = 3.09 \times 10^{-5} \pm 1.56 \times 10^{-5}$<br>$K = 0.00373 \pm 0.217$	0.996	6.90
	DDM	$D = 3.83 \times 10^{-15} \pm 5.36 \times 10^{-16}$<br>$U = 5.37 \times 10^{-11} \pm 2.85 \times 10^{-10}$	0.962	29.5
	DISEM	$U = 2.23 \times 10^{-12} \pm 3.47 \times 10^{-10}$<br>$C = 3.50 \times 10^{-5} \pm 2.95 \times 10^{-6}$<br>$K = 0.00372 \pm 0.363$	0.997	5.82
Cs_1_90	DSEM	$D = 1.40 \times 10^{-10} \pm 5.12 \times 10^{-11}$<br>$C = 0.00399 \pm 0.000551$<br>$K = 0.202 \pm 0.114$	0.966	13.0
	DDM	$D = 5.44 \times 10^{-10} \pm 7.70 \times 10^{-11}$<br>$U = 3.72 \times 10^{-10} \pm 2.56 \times 10^{-10}$	0.380	268
	DISEM	$U = 3.37 \times 10^{-7} \pm 7.04 \times 10^{-8}$<br>$C = 0.00598 \pm 0.000592$<br>$K = 0.0949 \pm 0.259$	0.906	20.3
Cs_3_90	DSEM	$D = 1.43 \times 10^{-10} \pm 4.75 \times 10^{-11}$<br>$C = 0.00451 \pm 0.000490$<br>$K = 0.183 \pm 0.0970$	0.974	20.2
	DDM	$D = 6.24 \times 10^{-10} \pm 1.07 \times 10^{-10}$<br>$U = 8.64 \times 10^{-11} \pm 2.65 \times 10^{-10}$	0.307	201
	DISEM	$U = 3.40 \times 10^{-7} \pm 7.24 \times 10^{-8}$<br>$C = 0.00651 \pm 0.000568$<br>$K = 0.101 \pm 0.248$	0.921	51.0

1.4 Residual Plots

1.4.1 Single source

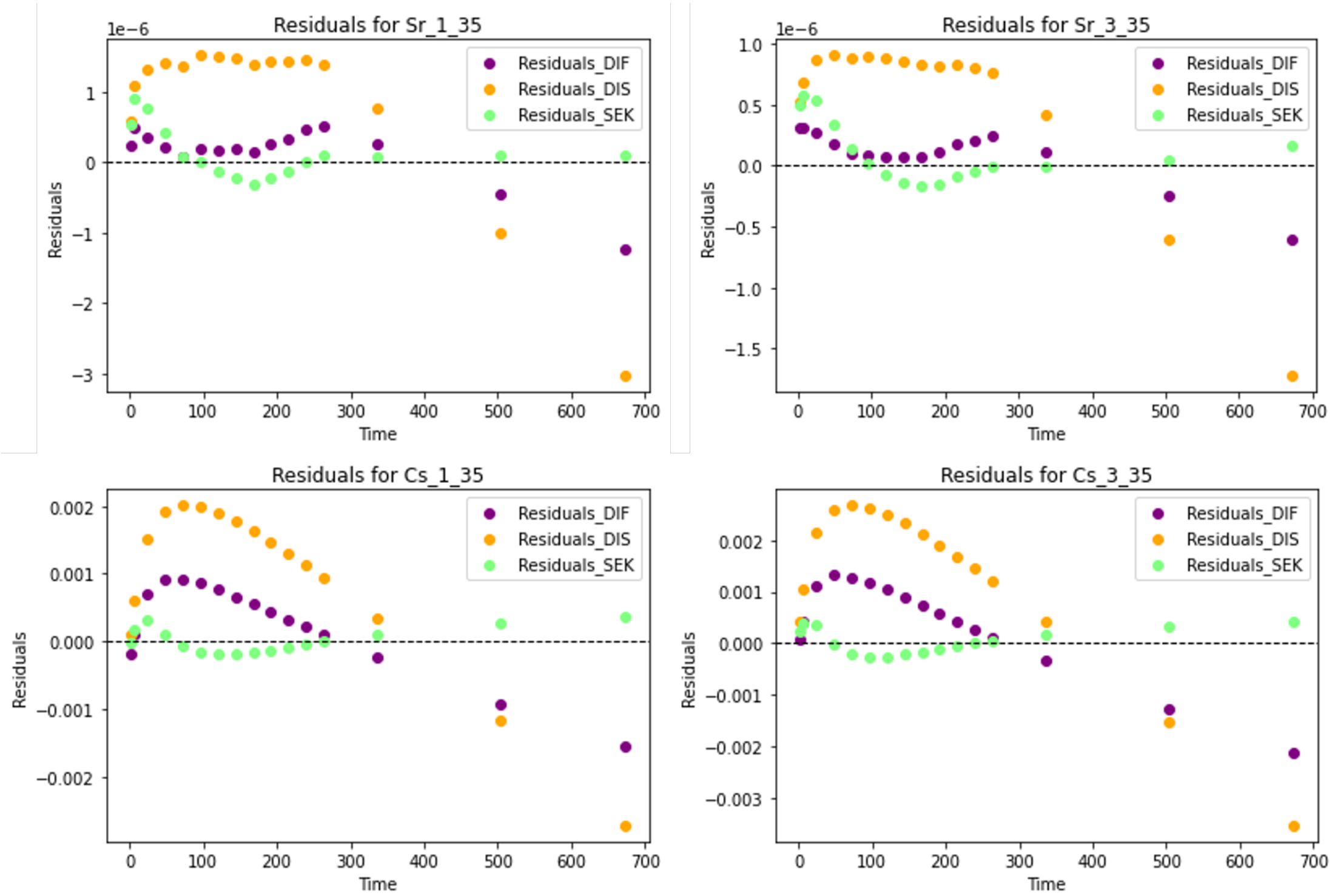

Figure 3: Residuals plots for the single source models from leach tests at 35 ° C.

1.4.2 Combined source

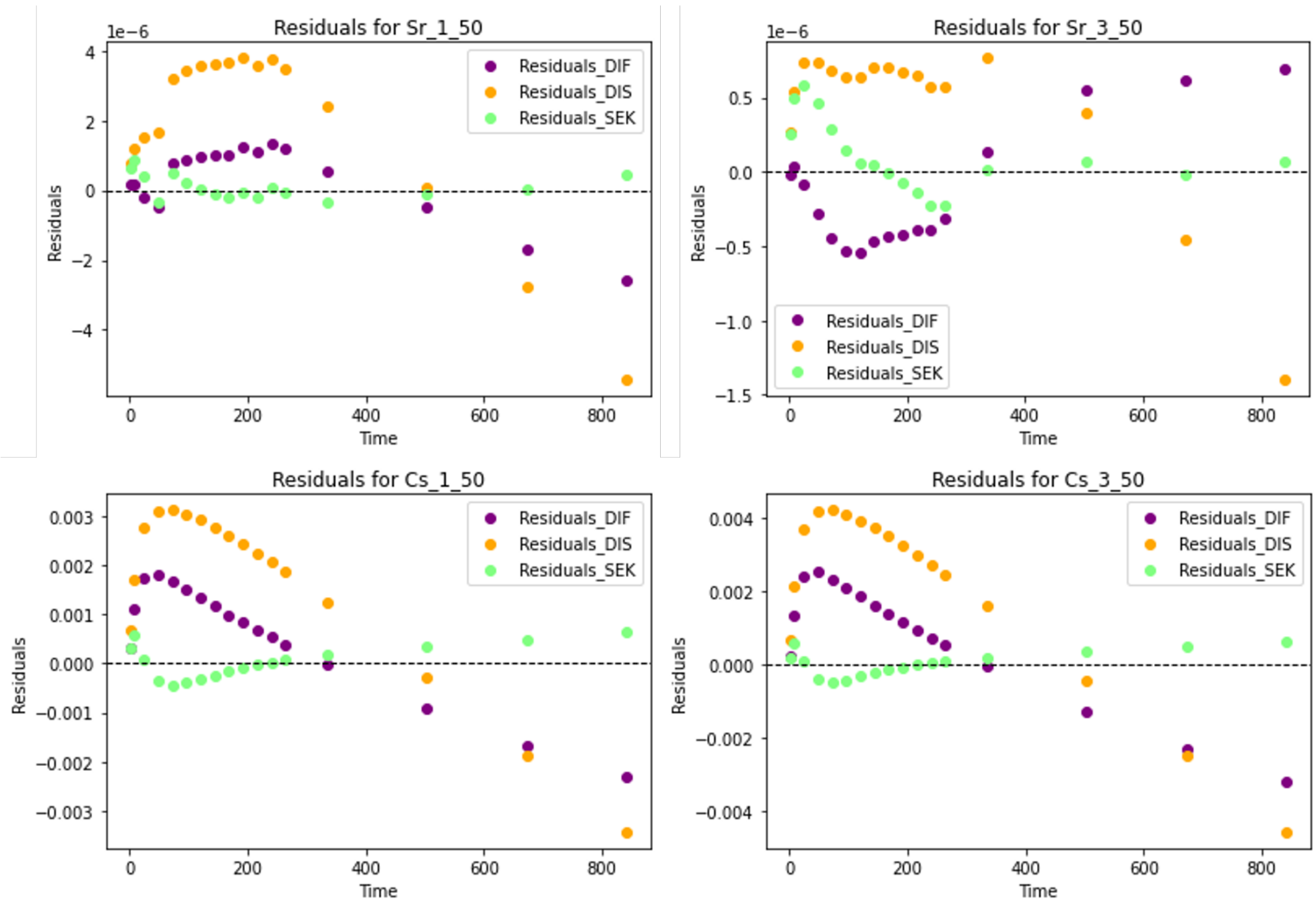

Figure 4: Residuals plots for the single source models from leach tests at 50 ° C.

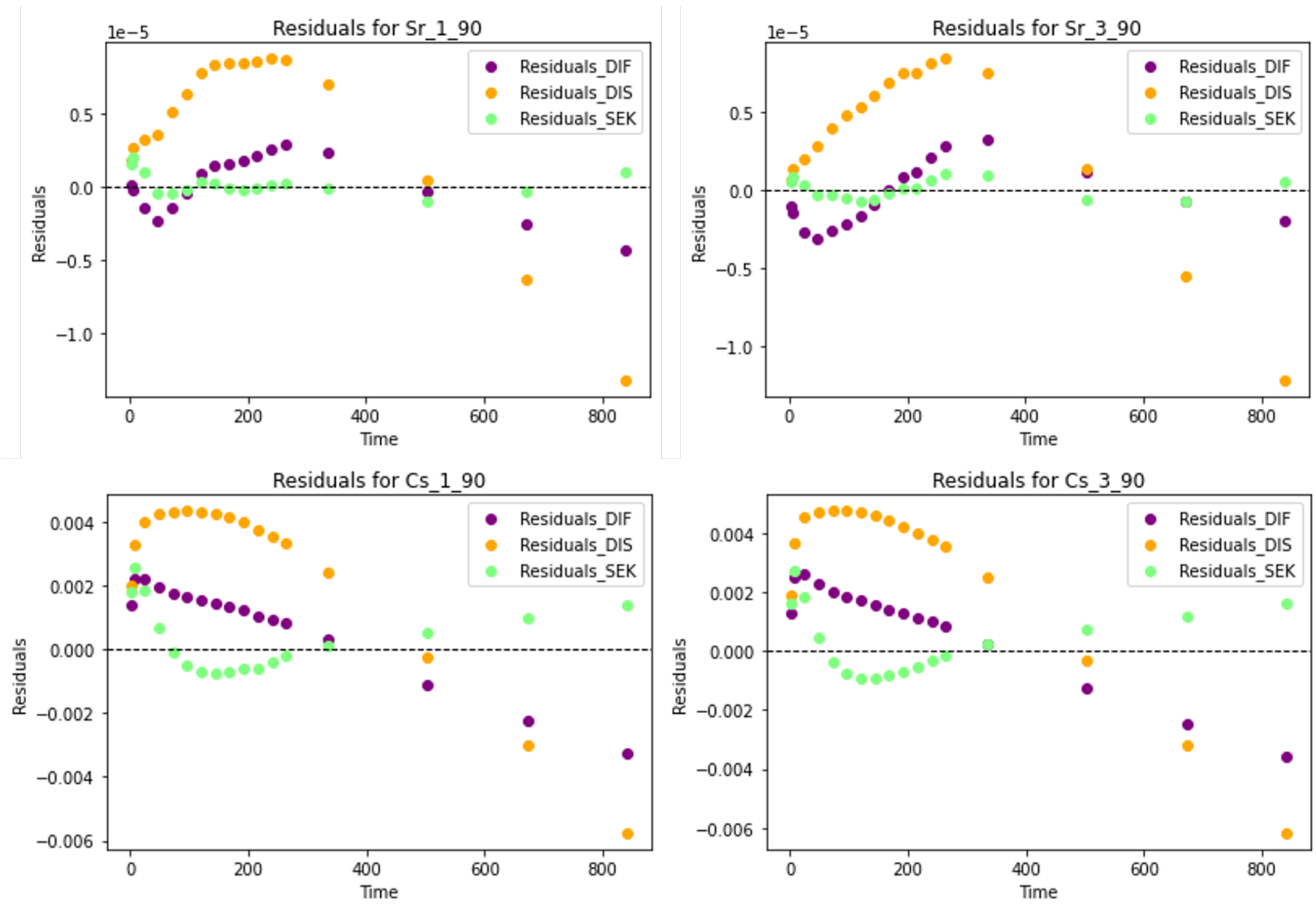

Figure 5: Residuals plots for the single source models from leach tests at 90 ° C.

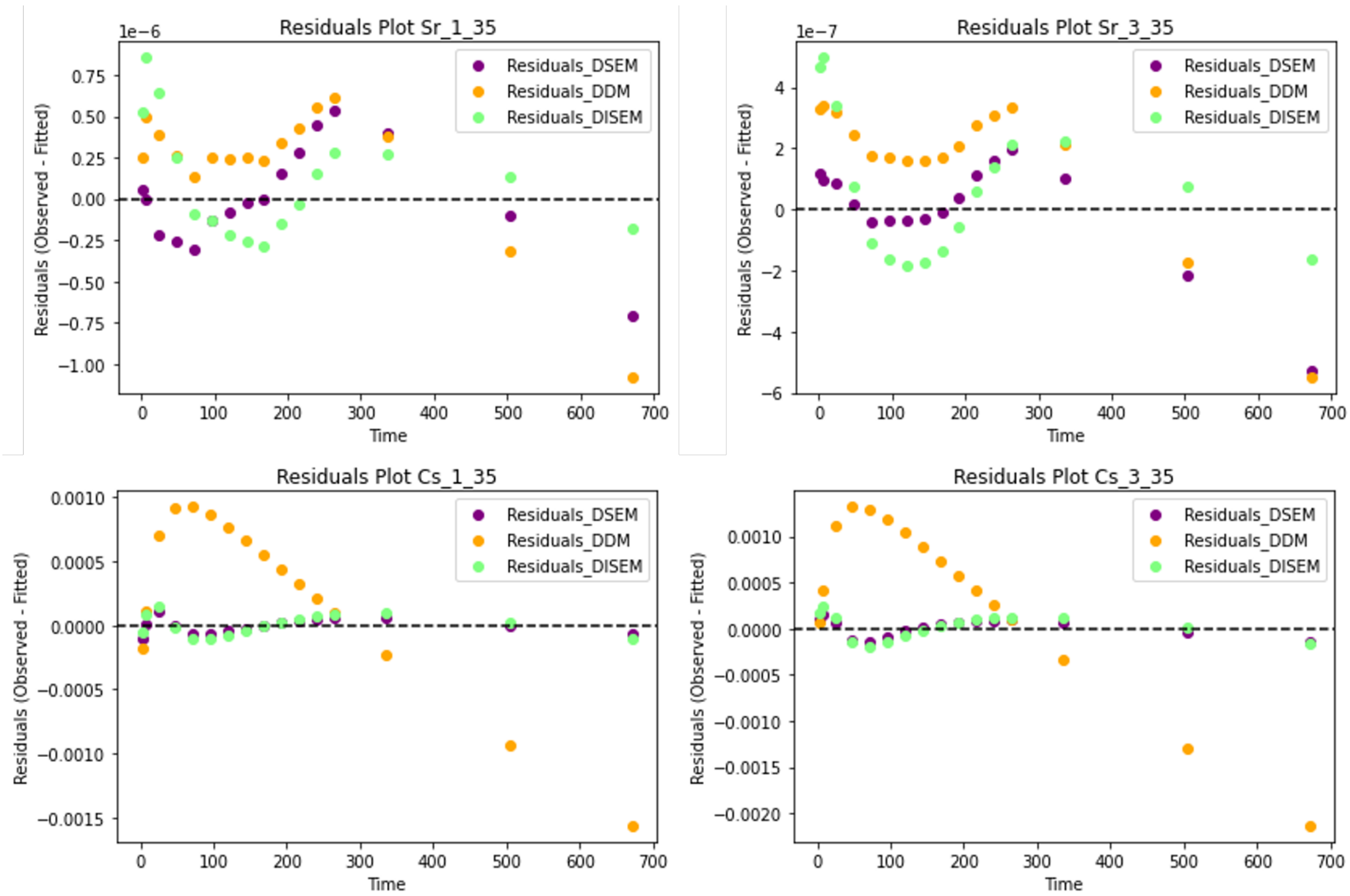

Figure 6: Residuals plots for the combined source models from leach tests at 35 ° C.

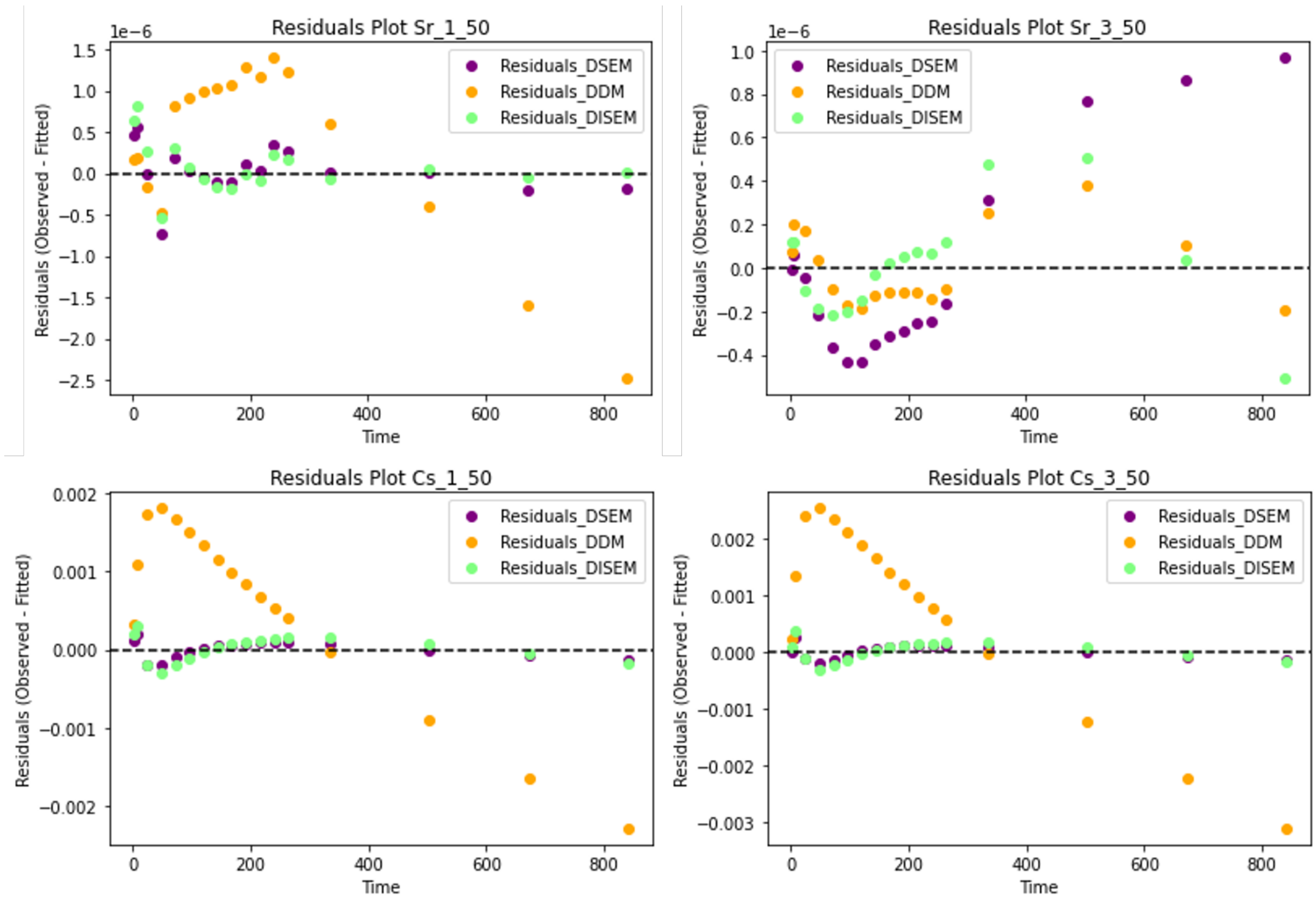

Figure 7: Residuals plots for the combined source models from leach tests at 50 ° C.

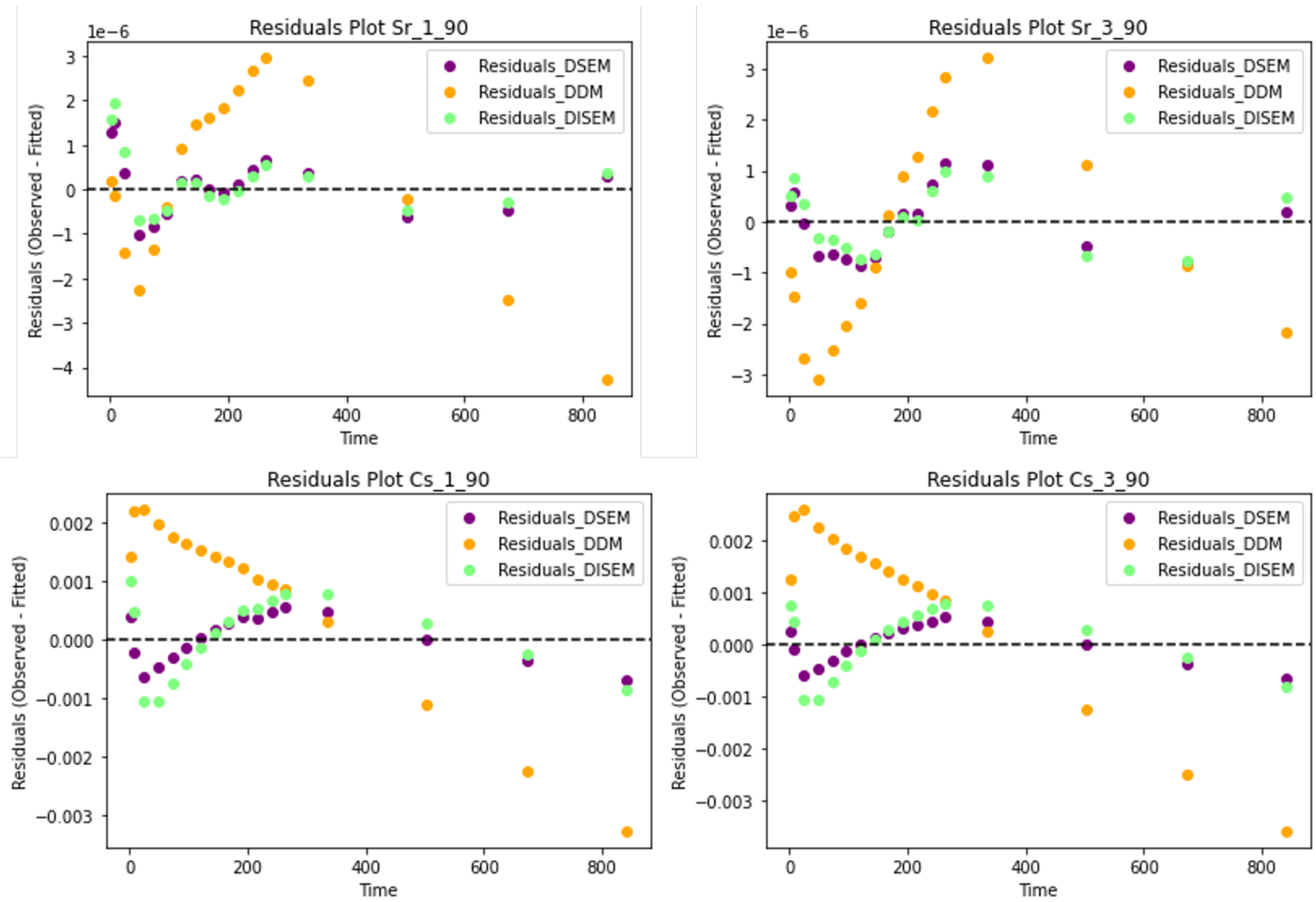

Figure 8: Residuals plots for the combined source models from leach tests at 90 ° C.
